# Modulation of optical absorption in m-Fe_1−x_Ru_x_S_2_ and exploring stability in new m-RuS_2_

**DOI:** 10.1038/s41598-021-86181-7

**Published:** 2021-03-23

**Authors:** H. Joshi, M. Ram, N. Limbu, D. P. Rai, B. Thapa, K. Labar, A. Laref, R. K. Thapa, A. Shankar

**Affiliations:** 1 Condensed Matter Theory Research Lab, Kurseong College, Darjeeling, 734203 India; 2 Department of Physics, St. Josephs College, North Point, Darjeeling, 734103 India; 3 Physical Science Research Centre, Pachhunga University College, Aizawl, Mizoram 796001 India; 4 Physics Department, Faculty of Science, King Saudi University, Riyad, Saudi Arabia; 5 Department of Physics, Mizoram University, Aizawl, 796009 India

**Keywords:** Materials science, Mathematics and computing, Physics

## Abstract

A first-principle computational method has been used to investigate the effects of Ru dopants on the electronic and optical absorption properties of marcasite FeS_2_. In addition, we have also revealed a new marcasite phase in RuS_2_, unlike most studied pyrite structures. The new phase has fulfilled all the necessary criteria of structural stability and its practical existence. The transition pressure of 8 GPa drives the structural change from pyrite to orthorhombic phase in RuS_2_. From the thermodynamical calculation, we have reported the stability of new-phase under various ranges of applied pressure and temperature. Further, from the results of phonon dispersion calculated at Zero Point Energy, pyrite structure exhibits ground state stability and the marcasite phase has all modes of frequencies positive. The newly proposed phase is a semiconductor with a band gap comparable to its pyrite counterpart but vary in optical absorption by around 10^6^ cm^−1^. The various Ru doped structures have also shown similar optical absorption spectra in the same order of magnitude. We have used crystal field theory to explain high optical absorption which is due to the involvement of different electronic states in formation of electronic and optical band gaps. Lӧwdin charge analysis is used over the customarily Mulliken charges to predict 89% of covalence in the compound. Our results indicate the importance of new phase to enhance the efficiency of photovoltaic materials for practical applications.

## Introduction

Transition metal dichalcogenides (TMDC’s) have gained extensive research interest in the field of material sciences due to their photocatalytic and high optical absorption which are beneficial for the development of high power photovoltaic solar cells^1^. Among the members of TMDCs, pyrite FeS_2_ (p-FeS_2_) looks promising not only because of its remarkable optical absorption coefficient (α≈10^5^ cm^−1^ in visible energy region) and photocurrent quantum efficiency (> 90%)^2,3^, but also due to its diverse features such as, suitable energy band gap ~ 0.95 eV, nontoxicity, cost-effectiveness and abundance^4–6^ in nature. Despite, several research works^7–9^ have reported suboptimal photovoltaic performance of p-FeS_2_ due to its low band gap. The reported band gaps were found to be 0.5 eV less than the optimum gap for solar cell applications as mentioned in the theory of Shockley and Queisser^10,11^. As a result p-FeS_2_ has been sidelined from the main technological research interest and has become famous as “fools gold” (mainly due to its texture and colour). Eventually, pyrite FeS_2_ has been successfully replaced by RuS_2_ due to its optimum band gap of 1.22 eV measured from optical absorption spectra^12^ and close resemblance of electronic structure. Nevertheless, the applications of p-RuS_2_ in photoelectrochemical and other energy related devices^13,14^ also made it a subject of huge interest. While, the optical absorption of p-RuS_2_ (10^3^ cm ^−1^)^12^ is 10^2^ times lower than that of p-FeS_2_. On contrary to photon absorption a sample of single crystal of p-RuS2 shows a narrow optical band gap^15^. Hence, this material was overlook by many researchers for nearly three decades, until Brunken et al.^16^ reported a high absorption coefficient (1.8 × 10^5^ cm^−1^) linked with thin film sample. On the other hand, the bulk sample failed to regain the research interest and to the best of our knowledge, only a handful of literatures are available on p-RuS_2_.

Interestingly, we report an uncommon and unknown orthorhombic structural phase of the compound, also known as the marcasite structure, which may come out as a potential solar cell material. Initially, the phase was considered to be an “undesired phase” for photovoltaic applications due to very low band gap value associated with it^17^, but recent theoretical studies suggest its importance due to compatible optical absorption coefficient and band gap as that of pyrite^18,19^. In the present report, we have studied the unique marcasite phase (m-phase) of RuS_2_ with energy band gap (1.3 eV) close to its pyrite phase and shows high optical absorption coefficient (10^6^ cm^−1^) outperforming RuS_2_ thin films, silicon and even direct gap materials such as GaAs. The orthorhombic structure of the compound was obtained by introducing new energy levels in the forbidden gap of parent m-FeS_2_ through Ru doping in Fe site. The corresponding orthorhombic phase was found to crystallize with space group *Pnnm* (No. 58) and classified as anomalous orthorhombic, like m-FeS_2_ analog. It is interesting and rather surprising to note that no available literature has considered Ru dopants as an efficient impurity to optimize the optical behavior of m-FeS_2_. Theoretical investigation by Sun and Cedar^20^ have shown Ru to be a feasible dopant in order to optimize the band gap for enhanced photovoltaic performance in p-FeS_2_. The same has been confirmed from experiment with enhanced band gap of the pyrite phase^21^. Although this shows that Ru acts as an effective dopant, many studies avoid extending the idea in understanding the effects in marcasite structure, indicating the approach to be problematic. It was noted that Ru dopant fails to increase the band gap of the parent m-FeS_2_ and therefore might have been considered ineffective for optimal photovoltaic applications. However, we show a high optical absorption to the order of 10^6^ cm^−1^ and is attributed to the Ru dopants. This is due to the involvement of different electronic states in the formation of electronic band gaps and optical gaps. Ru atoms additionally introduces a higher energy level due to 4*d* electron and therefore optical transition takes place between Ru 4*d t*_*2g*_ state in the valence band and Ru 4* deg* states in the conduction band, whereas electronic band gap is due to Ru 4d *t*_*2g*_ state and low lying S 3*p* state in the conduction band. Furthermore, the phase stability of the end product and doped alloys m-Fe_1-x_Ru_x_S_2_ (x = 0.25, 0.5, 0.75, 1.0) are also examined by adopting various stability conditions to further understand their technological applications. In this context, the mechanical stability described by their elastic constants, thermodynamic stability and the dynamical stability from the phonon dispersions are calculated. Our results question the literature regarding the low photovoltaic performance in marcasite phase and reveal the coexistence of m-and p-phase of RuS_2_ that may motivate the experimentalist for further verification.

## Methods

We report first principle investigations based on Kohn–Sham density functional theory (DFT)^22,23^, where the total energy is expressed in terms of electron density rather than the wave function. All-electron orbitals based full-potential (linearized) augmented plane-wave (FP-LAPW) method, as implemented in wien2k code^24^ is used for calculations. In FP-LAPW method, the unit cell volume is divided into the non-overlapping muffin-tin (MT) spheres centered at the atomic sites and the remaining interstitial region. Therefore, basis functions of two different sets are used to explain these regions. In the MT sphere region, the radial solution of the one-particle Schrödinger equation describes the basis set and is expanded into atomic orbitals while in the interstitial region, a plane-wave basis set is used. The electron exchange and correlation were treated within Perdew-Burke-Ernzerhof (PBE) generalized gradient approximation (GGA)^25^, with the following basis sets of valence states: Fe −*3p*^*6*^*3d*^*6*^*4s*^*2*^, Ru −*4p*^*6*^*4d*^*7*^*5s*^*1*^ and S −*3s*^*2*^*3p*^*4*^. This functional depends on the spatial charge density as well as the local charge density. To further investigate the choice of exchange correlation potential on the result sensitivity, the modified Becke Johnson (mBJ) potential is used for calculations, where the potential additionally depends on the kinetic energy density. The semi-local exchange potential imposed by mBJ potential is found to describe accurate band ordering and energy gaps, which is as precise as computationally demanding Green’s function screened Coulomb interaction calculations^26,27^. To account the dependence of photon absorption with electron correlation, calculations are performed with and without the inclusion of onsite Coulomb self-interaction parameter (U). First Brillouin-zone (IBZ) was integrated by Monkhorst–Pack scheme with optimized k-mesh of 21 × 17 × 27. Experimental verifications have shown that both FeS_2_ and RuS_2_ does not show magnetic ordering^28^, therefore to make our calculations consistent with the experimental scenario, constrained magnetic calculations were performed. The other necessary approximations and equations involved in the calculation are discussed in the supplementary information (SI) section.

## Results

### Phase transition and structural properties of the new phase

**Figure 1:**
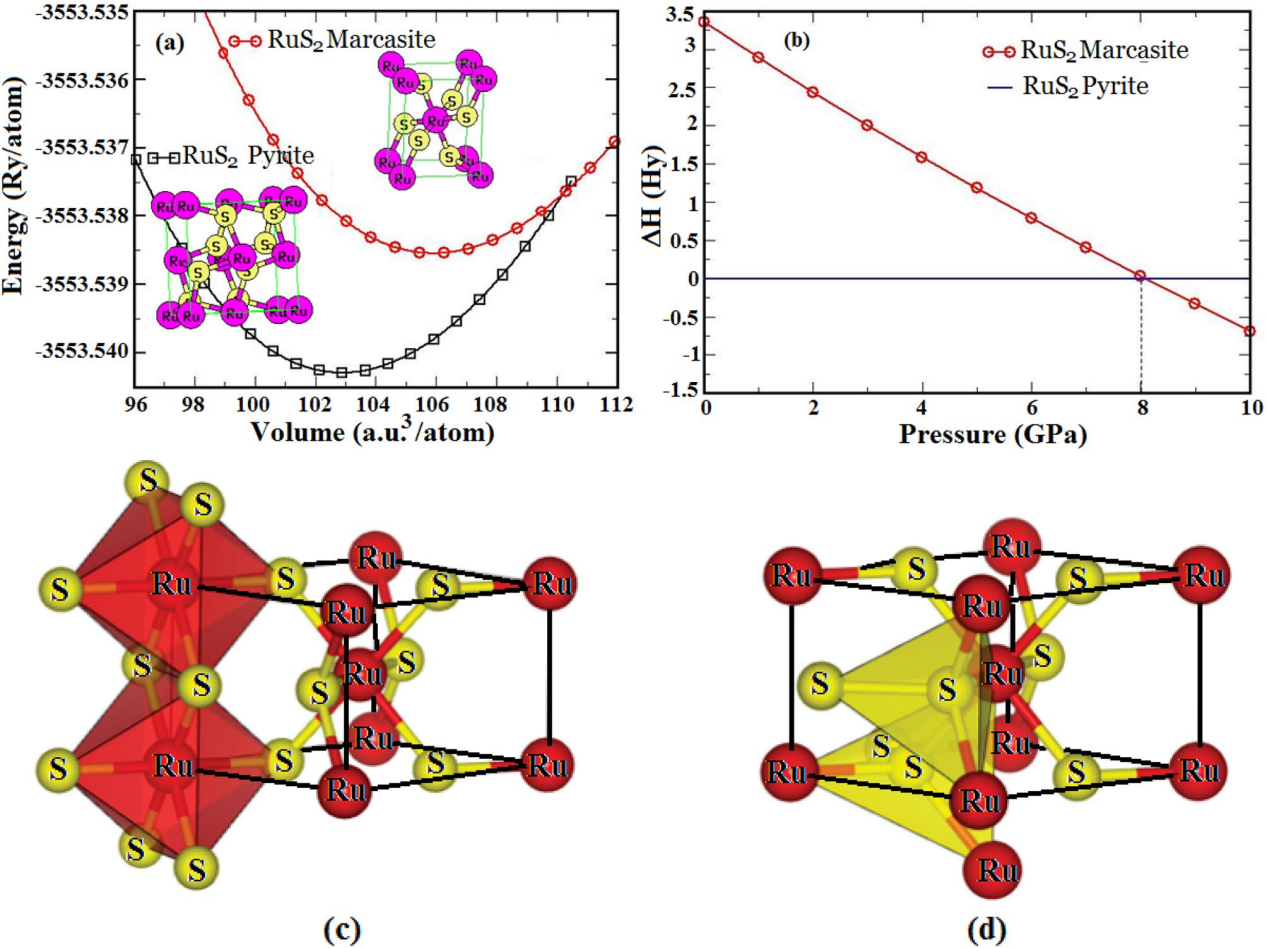
**(a)** Total energy curve as a function of relative volume. The red line with circle represents marcasite structure and the black line with square represents pyrite structure, **(b)** Phase Transition from Pyrite RuS_2_ to Orthorhombic RuS_2_. The dotted line perpendicular to X-axis denotes transition pressure, **(c)** The octahedral and **(d)** tetrahedral coordination of RuS_2_ orthorhombic phase.

We explore the possible orthorhombic marcasite structure of the compound obtained through a 2 × 2 × 2 supercell of m-FeS_2_ generating 16 Fe and 32 S atoms. When all Fe atoms are replaced by Ru followed by the space-group operation, leads to a m-RuS_2_ (space group *Pnnm*), a prototype of m-FeS_2_, generating six atoms per unit cell with two Ru and remaining S (Fig. 1a). Structural optimization with internal parameters 0.198 and 0.382 provides the optimized lattice constants of 4.6074 Å, 5.6641 Å and 3.6085 Å. Figure 1a shows the two optimization curves for pyrite and orthorhombic RuS_2_ fitted with Murnaghan’s equation of state and indicates p-RuS_2_ to be energetically more stable and naturally abundant in nature. The difference in enthalpy (∆H) plot between the two structures with respect to pressure (Fig. 1b) verifies the phase transition from pyrite structure of RuS_2_ with space group *Pa-3* to an orthorhombic structure with space group *Pnnm*. The transition phenomenon is calculated to occur at a pressure of 8 GPa and at this pressure, the conventional volume of pyrite and orthorhombic RuS_2_ are expected to be 172 Å^3^ and 90 Å^3^ respectively. However, p-RuS_2_ naturally occurs over a wide range of temperature and geological environment below 8 GPa and stable m-phase above transition pressure may open its applications in higher pressure range. In fact, the orthorhombic structure can be considered as the supergene origin of pyrite which is rare in nature.

The crystal structure of m-RuS_2_ is characterized by octahedral orientations of S atoms around Ru (Fig. 1c) and tetrahedral dimmers coordination of S atoms (Fig. 1d) like in p-phase. However, the octahedrons in the m-phase share their edges instead of their corner unlike in p-phase. The corner Ru atoms are surrounded by six S atoms from the neighboring cells which forms eight triangular faces (octahedron) and each such S atom has three Ru neighbors with an additional S forming a dimmer tetrahedron of S atoms. Therefore in essence, the orthorhombic RuS_2_ has similar crystal structure to that of m-FeS_2_. The Ru–S bond length of 2.37 Å in m-RuS_2_ is slightly extended as compared experimental Ru–S bond of 2.35 Å in pyrite phase^29^ and even Fe-S bond length of 2.23 Å in m-FeS_2_^30^. Likewise, the Ru–Ru bond length (3.61 Å) is wider than the Fe–Fe (3.38 Å) of m-FeS_2_. Furthermore, the S–S bond length (2.266 Å) in the tetrahedron dimer, calculated in terms of the internal parameters u and v (Eq. 1) is also higher as compared to p-RuS_2_ and m-FeS_2_.

1 $$ l_{S - S} \, = \,\left\{ {4u^{2} a^{2} + (1 - 2v)^{2} b^{2} } \right\}^{1/2} $$

The internal parameter which also measures the distortion from tetrahedral and octahedral geometry and v = 0.382 in m-RuS_2_ indicates the octahedral distortion in Ru sites with six neighboring S, where S–Ru–S bond angle is dilated to 98.96°. In addition, the dilated Ru–S-Ru bond angle of 119.18° is also the effect of the distortion of tetrahedral symmetry of S sites adjoined by three Ru atoms. The deviation of the lattice constants for the doped structures (Table 1 of SI) from a linear curve has been estimated by appending a bowing parameter (*b*_*p*_) in the Vegard’s rule, which is discussed insupplementary information (SI). We observed that the lattice parameters *a* and *b* have substantial deviation whereas *c* is almost linear, which is explained from their dynamical stability as discussed in later section. The pressure coefficients of *a*, *b* and *c* for m-RuS_2_ estimated from *P(M)* = {(*M*)^−1^*dM*/*dP*}_*P*=*0*_ are *P(a)* = 1.925 × 10^–3^ GPa, *P(b)* = 1.352 × 10^–3^ GPa and *P(c)* = 1.871 × 10^–3^ GPa, where *P(b)* < *P(c)* < *P(a)*, implies the least compressibility along *b*-axis and most along the *a*-axis. A similar observation has been reported in *m*-FeS_2_^31^ and the compressibility along $\overrightarrow{b}$-direction in RuS_2_ is even lower compared to FeS_2_. In general, the structural parameters of m-RuS_2_ show very low response to the external pressure and is accredited to the high bulk modulus of the compound, granting mechanical advantage to this material relative to its pyrite phase.

### Structural stability (formation energy)

The feasibility of the doping process in m-Fe_1-*x*_Ru_*x*_S_2_ (x = 0.25, 0.5, 0.75, 1.0) is verified from their formation energy (*E*_*f*_) and the calculation details are provided in supplementary information. The formation energy results are based upon DFT calculations performed at T = 0, P = 0 and therefore coincides with the enthalpy of formation. Lower *E*_*f*_ ensures the feasibility of doping in the host material and indicates thermodynamic stability and ease to synthesize experimentally. While growing the crystal the S-rich growth is much feasible, with low formation energy, over Fe-rich condition and has energy difference of − 5.72 eV between the two processes in case of RuS_2_ (Table 2 of supplementary information). The structure of the end product of the partial doping of m-FeS_2_ is independent of dopant (Ru) and it maintains the space group of P2/m (No. 10) except for 100% doping however, the *E*_*f*_ is inversely related to concentration of Ru and suggests high stability of m-RuS_2_. The observed formation energy of partially filled *4d*-Ru is high compared to *3d*-V, Cr, Mn, Co and Ni whereas lower than *4d*-Zr and Nb, that may persuade the *E*_*f*_ of m-RuS_2_ and the feasibility of the doping structure as compared to other dopants.

### Mechanical stability

The resistance of a material against a deformation can be understood from the elastic constants, which are the fundamental parameters to illustrate the structural stability and the anisotropic behavior of an alloy. The *m*-Fe_1-x_Ru_x_S_2_ has nine independent elastic stiffness constants, namely, *C*_*11*_*, C*_*22*_*, C*_*33*_*, C*_*44*_*, C*_*55*_*, C*_*66*_*, C*_*12*_*, C*_*13*_*, C*_*23*_*.* The estimated values of *C*_*44*_*, C*_*55*_ and *C*_*66*_ (trivial eigen values of this matrix) and *C*_*11*_*, C*_*22*_*, C*_*33*_*, C*_*12*_*, C*_*13*_ and *C*_*23*_ (eigen values of the cubic polynomial) satisfies the necessary and sufficient criteria (Eq. 2) that detail the orthorhombic crystal class with sufficient condition for mechanical stability^32^. The resistance to deformation along the non-axial directions is weaker than the axial directions over the entire concentration, where the *C*_*11*_, *C*_*22*_ and *C*_*33*_, which measure the resistance to linear compression, are much larger than the other elastic constants related to elasticity in shape.

2 $$ \begin{gathered} C_{11} \, > \,0,\,\,\,\,\,\,\,\,\,\,C_{11} C_{22} \, > \,C_{12}^{2} , \hfill \\ C_{11} C_{22} C_{33} \, + \,2C_{12} C_{13} C_{23} \, - \,C_{11} C_{23}^{2} \, - \,C_{22} C_{13}^{2} \, - \,C_{33} C_{12}^{2} \, > \,0, \hfill \\ C_{44} \, > \,0,\,\,\,\,\,\,\,\,\,\,\,C_{55} \, > \,0,\,\,\,\,\,\,\,\,\,\,\,C_{66} \, > \,0\, \hfill \\ \end{gathered} $$

The bonding along [010] is stronger than [001] and [100] directions with C_22_ > C_33_ > C_11_ for different values of *x* except 0.5, is consistent to the least and maximum compressibility along $\overrightarrow{b}$ and $\overrightarrow{a}$-directions, respectively as predicted in former section. The elastic response of polycrystalline *m*-Fe_1-x_Ru_x_S_2_ can be evaluated from the stiffness coefficient *C*_*ij*_*’*s and its derived parameters via Voigt (V) and Reuss (R) approximation^33^. The bulk modulus, which describes the resistance to volume deformation, obtained from the curve fitting method of Murnaghan equation of state (198.3 GPa) and elastic stiffness (201.8 GPa) are nearly equal that may validate the reliability of present elastic constants data. The end product of doping (RuS_2_) with x = 1 has the maximum resistance to volume deformation and is even higher than its pyrite phase (B = 133.3 GPa), whereas for x = 0.5 has least resistance. The hardness parameter (*HP*) of the compounds has also been investigated, which also shows m-RuS_2_ to be the hardest among all the compounds under consideration. Likewise, the capacity to resist the shape deformation (plastic deformation) against the applied hydrostatic pressure associated with shear modulus (*G*) is also high for m-RuS_2_ (118.5 GPa), which is even higher than its pyrite phase (G = 116 GPa). Furthermore, one can note a positive increment for G and Young’s modulus (Y) with x that can effectively enhance the resistance to shear deformation and stiffness, where RuS_2_ is stiffest of all that can be convenient for strong and flexible electronic applications. Similarly, Fe_0.75_Ru_0.25_S_2_ and RuS_2_ are ductile with G/B value within the critical limit^34^ of 0.57, which may be suitable in nano-technology due to their ability to deform without losing toughness, while the brittle nature can be expected for other values of x. The nature of ductility and brittleness can also be established from their Poisson’s ratio (η), which is inversely related to G/B ratio and denotes the central force nature of inter-atomic interaction with limiting values of 0.25–0.5. Also the closed packing of atoms in the RuS_2_ crystal can be inferred from high B as well as elastic wave velocities. The Debye temperature (*θ*_*D*_) also indicates the stability of these crystal structures and strong binding forces with high melting point. An elastically isotropic crystal has its anisotropy factor (*A*) value of unity and the deviation from unity measures the degree of anisotropy and probability of developing micro-cracks while growing a crystal. The m-RuS_2_ has three independent shear anisotropy factors *A*_*100*_, *A*_*010*_ and *A*_*001*_ with numerical values of 0.926, 0.772 and 0.80 along the {1 0 0}, {0 1 0} and {0 0 1} planes respectively. The values so obtained are in advantageous position with low degree of anisotropy in the structure than p-RuS_2_ ~ 0.5 in the crystal growth.

### Thermodynamic stability

**Figure 2:**
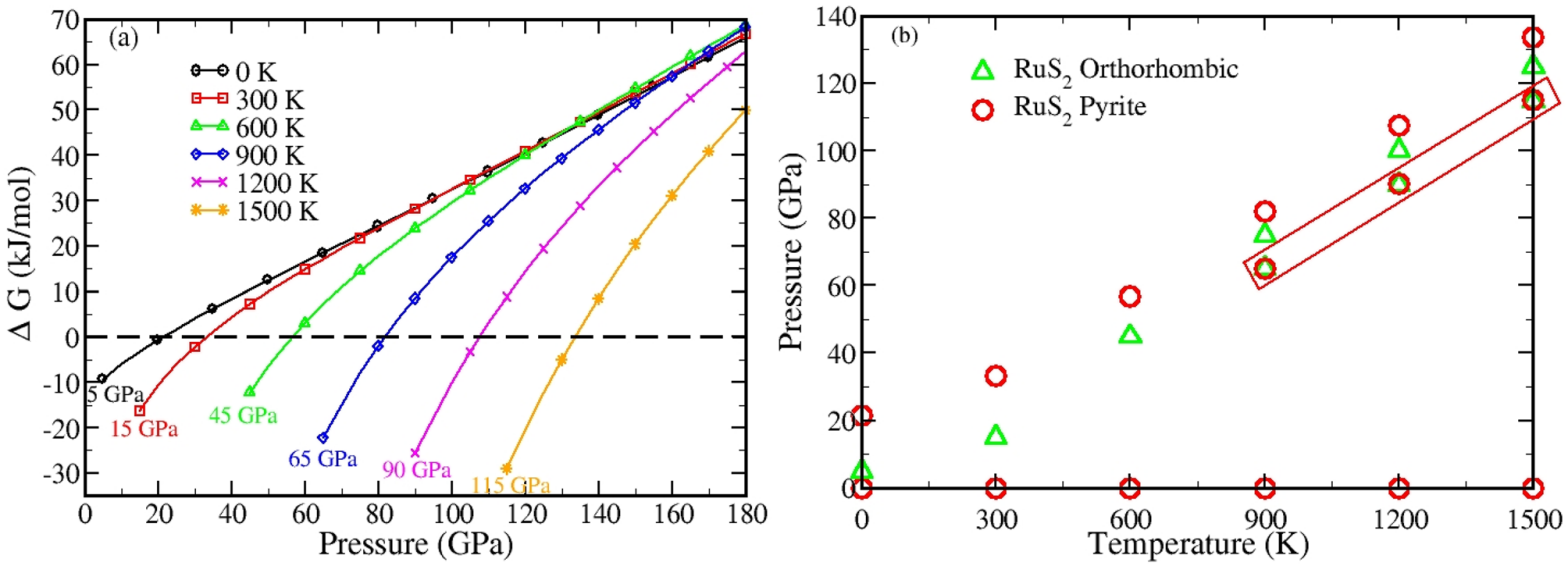
**(a)** Evolution of Gibbs free energy (∆G) with temperature and pressure for pyrite and orthorhombic phase of RuS_2_ **(b)** Phase stability in RuS_2_ as a function of temperature and pressure. The boxed region highlights the co-existence of both phases.

The class of semiconducting materials explored so far for commercial electronic and optoelectronic applications are also assess from their thermodynamic stability at the mechanical loading of wide range of temperature and pressure. Thus, the thermodynamic stability defines the equilibrium state of a material as well as the working environment, and also measures the existence of the system beyond equilibrium state with possibility of phase transition. The variation of thermodynamic parameter, such as Gibbs free energy (*∆G*) of m-phase with respect to p-phase for a wide range of temperature and pressure relates the feasibility of existence of m-phase in RuS_2_ (Fig. 2a). Here, the horizontal dotted line of ∆G = 0 denotes the thermodynamic equilibrium state and positive and negative ∆G for different temperatures and pressure indicates the stability of pyrite over orthorhombic phase and vice versa. At temperature of 0 K, ∆G is negative for a pressure range of 5 GPa to 21.5 GPa and at the upper limit of this pressure, the material is in equilibrium state. At other values of pressure, above and below the mentioned range, ∆G is again positive. Therefore, orthorhombic phase has the possibility to exist only between a pressure ranges of 5 GPa to 21.5 GPa at 0 K, whereas, pyrite is stable from 0 to 5 GPa and from 21.5 to 140 GPa at least (140 GPa being the highest pressure explored). However, the structural phase transition to orthorhombic does not take place at 5 GPa in case of RuS_2_ due to the reasons discussed in the following section. Similarly, for other values of temperature, the least value of pressure for the orthorhombic phase to exist is 15 GPa at 300 K, 45 GPa at 600 K, 65 GPa at 900 K, 90 GPa at 1200 K and 115 GPa at 1500 K. The orthorhombic phase stability can further be analyzed from Fig. 2b, where the existence of different phases for wide range of temperature and pressure are shown. The pressure stability range corresponding to different temperature for different phases as revealed from Fig. 2b are unanimous with the previous section results, where we report the pyrite phase to be the ground state phase in RuS_2_.

For instance, at temperature 900 K and at a pressure below 65 GPa, only the pyrite phase can exist, as this phase is the only stable one in the range. However at 65 GPa, the orthorhombic phase also becomes stable and both the phases are predicted to co-exist at this pressure value (boxed region in the figure). The co-existence can continue up to a maximum of 75 GPa and above this pressure value, the orthorhombic phase surpasses the stability of pyrite until 81.8 GPa pressure is reached, beyond which pyrite dominates the existence up to further pressure. Temperatures of coexistence, other than that shown in the figure are 550 K, 800 K, 850 K, 950 K and 1300 K. It is to be noted that at pressure values achieved naturally, the pyrite stability dominates the marcasite (for all temperature values), which can exist only at a limited range of pressure and can be considered as the reason why the orthorhombic phase remained hidden. Likewise, the negative ∆G and the phase stabilities are also observed for other concentration (0.25, 0.5 and 0.75) of x and they are further analyzed from the variation of their specific heat at constant volume (C_v_) with respect to pressure (see supplementary section). The phase stability of FeS_2_ is already established in previous literature hence x = 0 is not calculated here.

### Dynamical stability and mechanism for phase transition

**Figure 3:**
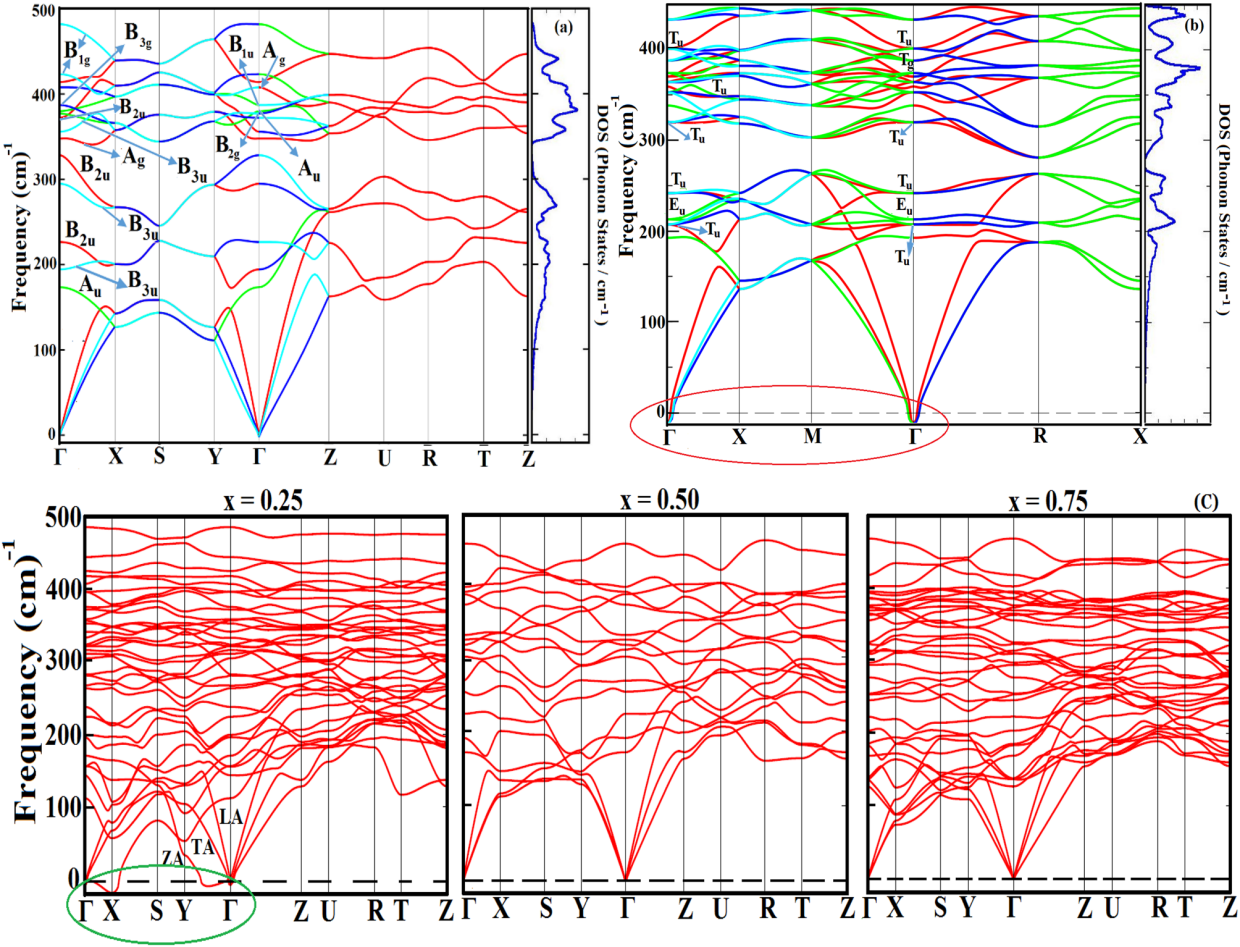
Zone-centered phonon vibrational frequencies and symmetries of different optical modes (marked by arrows) of **(a)** orthorhombic RuS_2_ and **(b)** pyrite RuS_2_ at 8 GPa along with the total phonon density of states. **(c)** Phonon dispersion curves for m-Fe_1−x_Ru_x_S_2_. The green circle marks the negative frequencies along the xy-plane. In the figure, LA, TA and ZA denotes longitudinally acoustic, transversely acoustic and out of phase acoustic modes, respectively.

The phonon dispersion curves were calculated by diagonalization of Monkhorst − Pack grid of 4 × 4 × 4 k-points corresponding to phonon wave vectors and set up for dynamical matrices of *m*-Fe_1-x_Ru_x_S_2_ (x = 0.25, 0.5, 0.75, 1.0) at ambient conditions. The phonon dispersion curve for the entire first Brillouin zone has been investigated (Fig. 3) to analyze the every single phonon mode that would compromise the dynamical stability of the sample alloys with imaginary frequency. The vibrations are non-degenerate type at *Γ*-point that undergoes two-fold degeneracy along the high symmetrical *Y–Γ–Z* direction. The figure also confirms the dynamical stability of the orthorhombic phase with no negative frequencies at the zone centers. Interestingly, phonon softening arises in the pyrite phase at 8 GPa pressure (Fig. 3b) that confirms the phase transition in RuS_2_. The vibrational mode at Γ-point gradually softens with the increase in pressure and eventually becomes negative at a pressure of 8 GPa. The presence of the imaginary frequency in the dispersion curve supports the instability of the pyrite *Pa-3* structure and hence the structure is transformed to a new one. The triply degenerate acoustic *T*_*u*_ vibrational mode in pyrite RuS_2_ can be accredited for phonon softening and the corresponding phase transition. The number of Raman (R) and Infrared (IR)—active modes and their respective symmetries are determined from group theory analysis (see Supplementary Information Section) and due to inversion symmetry of RuS_2_ crystal, the R and IR modes are mutually exclusive modes. We have predicted the pyrite type ground state of RuS_2_, however there also exist discrepancy in between the experimental and PBE-GGA based theoretical studies on analogous FeS_2_^35^. Phonon spectra studies of the analogous compound by Spagnoli et al. reported a marcasite type of ground state, whereas the possible pyrite phase from PBE-GGA may be due to the effect of zero point energy (ZPE). Hence, the phonon spectra of p-RuS_2_ is also analyzed (Table 4 of SI) to compare the ZPE with m-phase using Eq. (3) to further verify the results of PBE-GGA.

3 $$ E_{0} \, = \,\sum\limits_{i\, = \,4}^{N} {\omega_{i} \,\left( {\frac{\hbar }{2}} \right)} $$

Here, *ω* is the vibration frequency, *ћ* the reduced Plank’s constant and *N* is the number of modes. The symmetries of IR and Raman R active optical modes with irreducible representation of vibrations of pyrite symmetry is expressed as

4 $$ \Gamma \, = \,A_{g} \, + \,E_{g} \, + \,3T_{g} \, + 2A_{u} \, + \,2E_{u} \, + \,6T_{u} $$

Here, 12 atoms per unit cell of p-RuS_2_ correspond to 36 phonon modes. One should note that E’s are doubly degenerate and T’s are triply degenerate modes. The ZPE difference between the two phases (0.036 eV) is in close agreement with PBE-GGA estimated value of 0.038 eV. The conventional DFT as well as phonon frequencies are close enough to establish the relative stability of two phases of ruthenium disulfide where ZPE values are compatible with GGA predicted ground state stability of pyrite over the orthorhombic.

The analysis of figures (Figs. 1, 2 and 3) and the stability criterion discussed in former sections imply the coexistence of p and m-phase of RuS_2_. Moreover, PBE-GGA and ZPE study reveal the stability of pyrite phase over marcasite that requires at least an isotropic pressure of 5 GPa to rupture the structure to other phase, pressure being a promising factor towards transition. Besides that owing to hard and brittle nature of m-RuS_2_, the atomic spheres are not exactly interchanged with the neighbors. However, the external stress or temperature induces a homogenous atomic movement through subtle atomic arrangement that evolves a first order solid–solid diffusion less phase transition also known as martensitic transitions^36^. In fact, this rearrangement can be described as a degradation of the highly symmetric cubic lattice into orthorhombic one with different variants of orthorhombic Bravais lattices. Since, the phase transition is driven by external factor (pressure, temperature), we first assume the transition from ordered to disordered cubic structure associated with the mobility of S atom, which is abundance in the cubic structure.

This gives rise to the order–disorder transition due to the inversion of the Ru–S unit cubic phase because of the random orientation of RuS_6_ octahedral and the disorder in the migrated S atoms. This transition is expected to withstand the pressure in the range of 5–8 GPa and the inversion of the Ru–S units is also responsible for the pressure induced cubic orthorhombic transition. Now, for the further increase in pressure, the Ru–S bonds breaks thus detaching the associated S atoms making free to move into new sites that leads to ordered orthorhombic state. In the cubic structure, Ru atoms lie along the < 111 > three-fold axis of the unit cell and three of such Ru atoms bound two S atoms from the tetrahedral surrounding. While, in transition from cubic to orthorhombic structure, the terminal S atoms are rearranged, such that half of the total of such Ru–S tetrahedral units gets reversed in direction. This inversion of half of the tetrahedral units eventually induce the positioning of two Ru atoms and four S atoms outside the unit cell, reducing the number of atoms and thus the volume of the orthorhombic structure to half of the cubic cell. The Ru–S tetrahedral units tilt off the original three fold axis forming a two-fold symmetry axis pointing along the z-direction in such a way that the terminal S atoms get associated with the neighboring Ru. In fact, the volume collapse of 3.4% is due to the topological changes in the Ru–S sublattices made possible by this tetrahedral tilts and the degree of tilting can be characterized by Ru–S-Ru angles, which is greater in orthorhombic (119.8^0^) as compared to the regular pyrite structure (115.0^0^). Similarly, the S–Ru–S bond angle in orthorhombic (98.96^0^) is also greater than in pyrite (94.1^0^). However, in spite of this expansion in Ru–S bond angles, the Ru and S atoms in the orthorhombic phase do not move significantly apart. The Ru–S bond length in cubic, which is 2.35 Å^29^, is almost same as the calculated bond length of 2.37 Å in orthorhombic (only 0.8% longer). It may be also mentioned here that the Ru–S bonding dominates the *b* lattice parameter and will remain almost same as that in pyrite. The transformation of crystal structure due to change in S–S bonding (compression of lattice constants *a* and *c*) also explains the hardness of the material and increase in resistance against compression along the $\overrightarrow{b}$-direction. Since, the volume of the crystal and the number of atoms in the m-RuS2 reduces to half of that in pyrite phase, it can be considered as the splitting of pyrite unit cell into two orthorhombic cells sharing a common plane as a habit plane where the lattice deformation is given by the distortion matrix^37^

5 $$ D\, = \,\left( {\begin{array}{*{20}c} {\varepsilon_{1} } & 0 & 0 \\ 0 & {\varepsilon_{2} } & 0 \\ 0 & 0 & {\varepsilon_{3} } \\ \end{array} } \right) $$

where, $\varepsilon_{1}$_,_ $\varepsilon_{2}$ and $\varepsilon_{3}$ are the principle lattice distortion calculated from the lattice parameter of the parent and the product phase as $\varepsilon_{1} \, = \,\frac{{a_{0} }}{{a_{c} }}\, - 1$, $\varepsilon_{2} \, = \,\frac{{b_{0} }}{{a_{c} }}\, - 1$ and $\varepsilon_{3} \, = \,\frac{{c_{0} }}{{a_{c} }}\, - 1$ and knowing these distortion, various crystallographic parameters can be obtained. The habit plane and the orientation relationship of [001], [010] and [100] directions between the disordered cubic and ordered orthorhombic phase as calculated from Eqs. (6) and (7) is along [0.998 0.06 0] is close to [1 0 0] direction.

6 $$ Habit\,Plane\,\, = \,\,\left[ {\left( {\frac{{\varepsilon_{1} + \varepsilon_{3} }}{{\varepsilon_{1} + \varepsilon_{3} - \varepsilon_{2} }}} \right)^{1/2} \,,\, - \left( {\frac{{\varepsilon_{2} }}{{\varepsilon_{1} + \varepsilon_{3} - \varepsilon_{2} }}} \right)^{1/2} \,,\,0} \right] $$

7 $$ \begin{gathered} \left[ {100} \right]_{\,c} \, \wedge \,[100]\,_{o} \, = \,\left[ { - \varepsilon_{2} \,(\varepsilon_{1} \, + \,\varepsilon_{3} ) + \varepsilon_{3}^{2} } \right]^{1/2} \hfill \\ \left[ {010} \right]\,_{c} \, \wedge \,[010]\,_{o} \, = \,\left[ { - \varepsilon_{2} \,(\varepsilon_{1} \, + \,\varepsilon_{3} )} \right]^{1/2} \,\,\,\,\,\,\,\,\, \hfill \\ \left[ {001} \right]_{\,c} \, \wedge \,[001]\,_{o} \, = \,\left| {\varepsilon_{3} } \right| \hfill \\ \end{gathered} $$

The disordered cubic and ordered orthorhombic has orientation relationship such that their principle axis are almost parallel. These are $\left[ {100} \right]_{\,c} \, \wedge \,[100]\,_{o}$ = 0.362, $\left[ {010} \right]_{\,c} \, \wedge \,[010]\,_{o}$ = 0.03 and $\left[ {001} \right]_{\,c} \, \wedge \,[001]\,_{o}$ = 0.361. Finally, the obtained orthorhombic structure is classified as anomalous orthorhombic, depending upon the c/a ratio (> 0.57) and Ru–S-Ru bond angle (> 90^0^).

In addition, the phonon dispersion curve for the doped structure (m-Fe1-xRuxS2, x = 0.25, 0.5, 0.75) is also evaluated (Fig. 3c) to understand their dynamical stability. The negative frequencies along *Γ-X* and *Y-Γ* symmetry directions for x = 0.25 is a signature of instability of *P2/m* phase that may go into phase transform in consistent to formation energy that also indicates the least stability. In this structure the softening of the phonons occurs due to the hybridization of out-of-plane vibrations of the acoustic mode (ZA) (ZA corresponds to the out of phase acoustic vibration) with respect to the xy-plane in the crystal, where the high symmetry lines *Γ–X* and *Y–Γ* lie with the in-plane vibration along Z-axis. The PBE-GGA functional usually overestimate the calculation by predicting the lattice parameters *a* and *b* beyond the acceptable range, leading to unphysical phonon dispersion along the x-and y-axes. While, the in-plane parameter *c* remains exempted from overestimation and hence no negative frequencies are observed along the z-axis. This readily explains the high bowing of *a* and *b*, whereas almost no bowing of the parameter *c* with Vegard’s rule. However, it is to be emphasized that no phonon softening at the zone-centers are found for Ru concentrations of 0.5 and 0.75, which still questions the validity of the rule in these alloys. The absence of phonon band gap in the phonon spectrum of these alloys indicates a constant scattering among the optical and the acoustic branches, and therefore a low thermal conductivity of these compounds can be expected.

### Nature of bonding and origination of band gap in RuS_2_ marcasite phase

**Figure 4:**
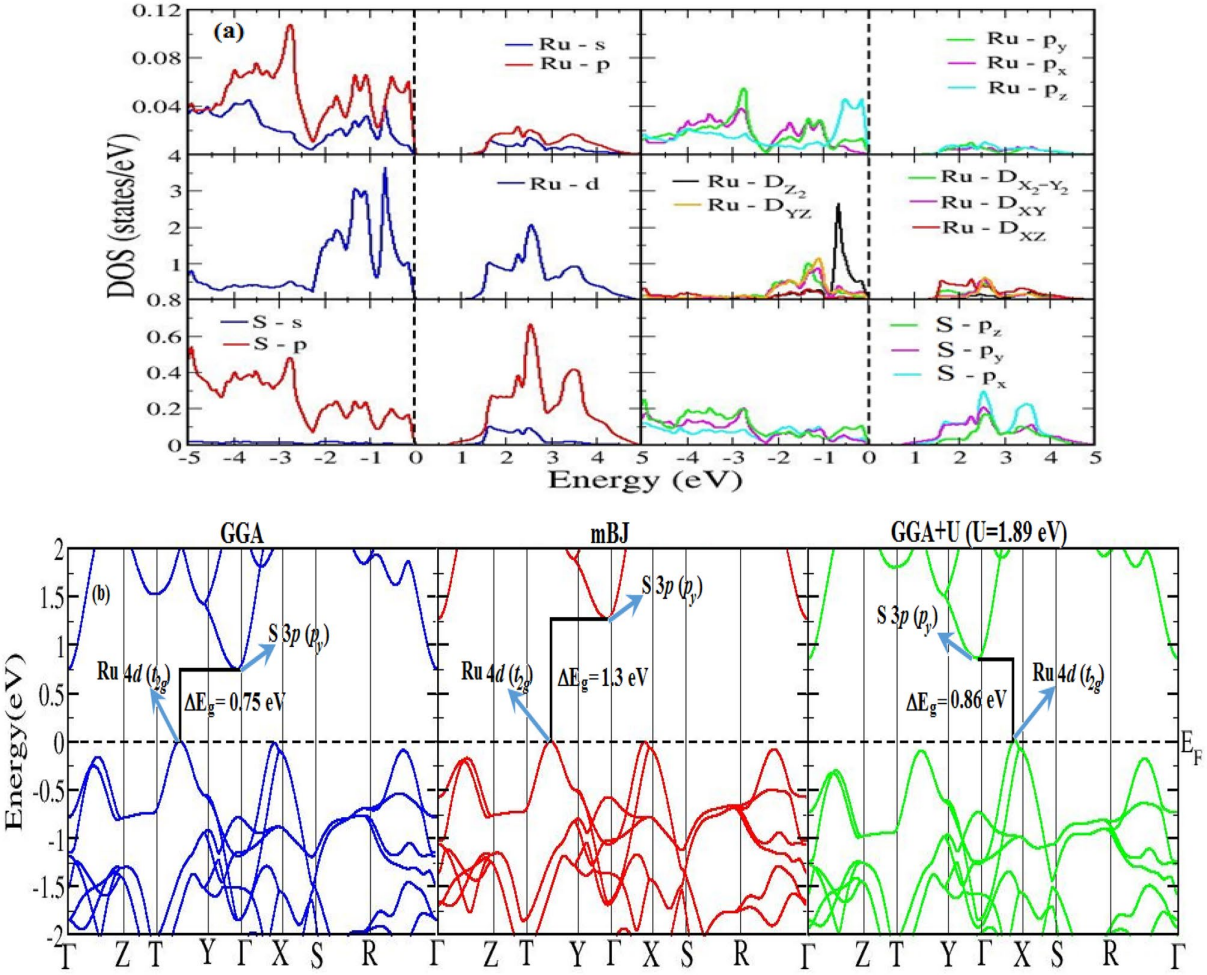
**(a)** Partial density of states for marcasite RuS_2_, showing different orbital contribution in the valence and conduction region. **(b)** Energy band structure of marcasite RuS_2_, with dominant orbital characteristics in the valence and conduction band edges. Note the shift in VBE under GGA + U scheme.

A semiconducting nature of moderate energy band gaps of 0.95 eV, 1.17 eV and 1.22 eV^11,12,20^ for marcasite and pyrite phase of FeS_2_ and p-RuS_2_, respectively have been widely explored. Moreover, the addition of impurity atoms such as V, Cr, Mn, Co, Ni, Cu and Zn on m-FeS_2_^38^ also produces significant effect with modified band gap semiconductor or the half metallic end product. In line to them, in the present study, the nature of variation of energy band gap of m-Fe_1-x_Ru_x_S_2_ is studied to predict the suitable composition for photovoltaic applications. It is known that the traditional PBE-GGA overestimate the calculation by predicting underestimated electronic band gap for semiconductors and insulators about 30–40% of experimental report^39^. This usually happens due to self-iteration and GGA does not precisely account for the quasiparticle self-energy and hence lacks in derivative discontinuities of the exchange correction in terms of occupation number. In such circumstances, the modified Becke-Johnson potential (TB-mBJ) within the parameterization of Trans and Blaha^40^ is also efficient while correcting band gaps close to experimental data for semiconductors. Note here, the sample alloys possess highly correlated transition metals with localized *d*-orbitals poorly described by PBE-GGA. The Hubbard *U* correction term is introduced to guarantee the spatial distribution of the partially filled *d*-orbitals to the GGA calculations (GGA + U), which combine the explicit treatment of electronic correlation by introducing onsite Coulomb self-interaction potential (*U*). The electronic structure of a system is sensitive to the system dependent *U* and hence optimized in the present calculation for Fe-*3d* and Ru-*4d* states as *U*_*eff*_ = 2 eV and 1.89 eV, respectively. The electronic properties explained by density of states (DOS) and energy band structures under different approximations as discussed above are presented in the supplementary information section. The localized *d*-electronic states dominate the DOS characteristics (Fig. 4a), which imposes the restriction to the number of valence electrons of the *d*-states thereby reducing its energy as well as occupation and hence the amplitude of peaks is diminished in GGA + U (Figure in supplementary information section). Furthermore, the reduced occupation also leads to abrupt reduction in total DOS and also the energy of valence electrons, thereby improving the magnitude of band gap (*E*_*g*_). The effective mass of the conduction band edge increases on choosing mBJ potential (except x = 0.25), thereby making them flatter and less dispersed. This is expected as the mBJ potential treats the valence electrons semi-locally that weakens the orbital interaction resulting in high effective mass. The exception on x = 0.25 attributed to the structural instability. The GGA calculation of m-RuS_2_ predicts the E_g_ values of 0.75 eV, which is considerably underestimated as compared to its pyrite structure of 1.22 eV. This underestimation by GGA is associated with the positioning of the Ru *4d* bands at low binding energy with respect to the valence band edges. The underestimated value has been enhanced significantly to 1.3 eV with the inclusion of mBJ, as expected, and is considered as the standard E_g_ values of the proposed phase. It is also to be noted here that the GGA + *U* evaluated E_g_ strongly depends on *U*_*eff*_ value and E_g_ = 1.3 eV can be acquired with *U*_*eff*_ = 10–12 eV. However, the structural parameters are poorly described by this *U*_*eff*_ value. Hence we obtain E_g_ for pyrite and marcasite RuS_2_ close to each other with mBJ, which is of particular interest for considering the photovoltaic purpose of the marcasite phase.

The fundamental band gap in m-RuS_2_ is due to the difference of VBM lying between *T–Y* symmetry direction (*H* symmetry point) and CBM at *Γ* point, with conduction and valence band edges traced to S-*3d* and Ru-*4d* dominant character. The overall profile of energy bands and DOS is similar for pyrite phase except the high contribution of S-*3d* and Ru-*4d* in pyrite structure and the VBM being positioned elsewhere. Likewise, the orbital character of electronic bands are also similar in m-FeS_2_ and p-RuS_2_, except the dispersed nature of conduction and valence bands of p-RuS_2_ and the position of FeS_2_ CBM at symmetry point other than *Γ*-point.

Figure 4b displays the band structure of marcasite RuS_2_ under different schemes. The orbital contribution remains significantly same on choosing any of GGA, mBJ or GGA + U methods, however, the band characteristic exhibits major changes with GGA + U result. The inclusion of Hubbard parameter (U) corrects the Kohn–Sham energies and thus the Ru 4*d* state at the band edges is shifted considerably, whereas the S 3*p* state at Γ-point is only corrected slightly. Overall, the energy gap at the band edges is only slightly corrected and the valence band edge (VBE) observed at *H*-symmetry with PBE-GGA is shifted near to X. VBE at this symmetry point shows mixed characteristic with considerable contributions from Ru *p*_*z*_, S *p*_*z*_ and Ru *d*_*z2*_, however, Ru *dt*_*2g*_ dominates the highest occupied VBE touching the Fermi level. The band gap increases from 0.75 to 0.86 eV and is observed between *Γ*-symmetry point in the conduction band and symmetry point lying close to *X* in the valence band which is due to the slight change in hybridization with the S 3*p* state at *Γ*-point. Although, the energy band gap is significantly improved, the optical property worsens with GGA + U and is discussed in the following section.

**Figure 5:**
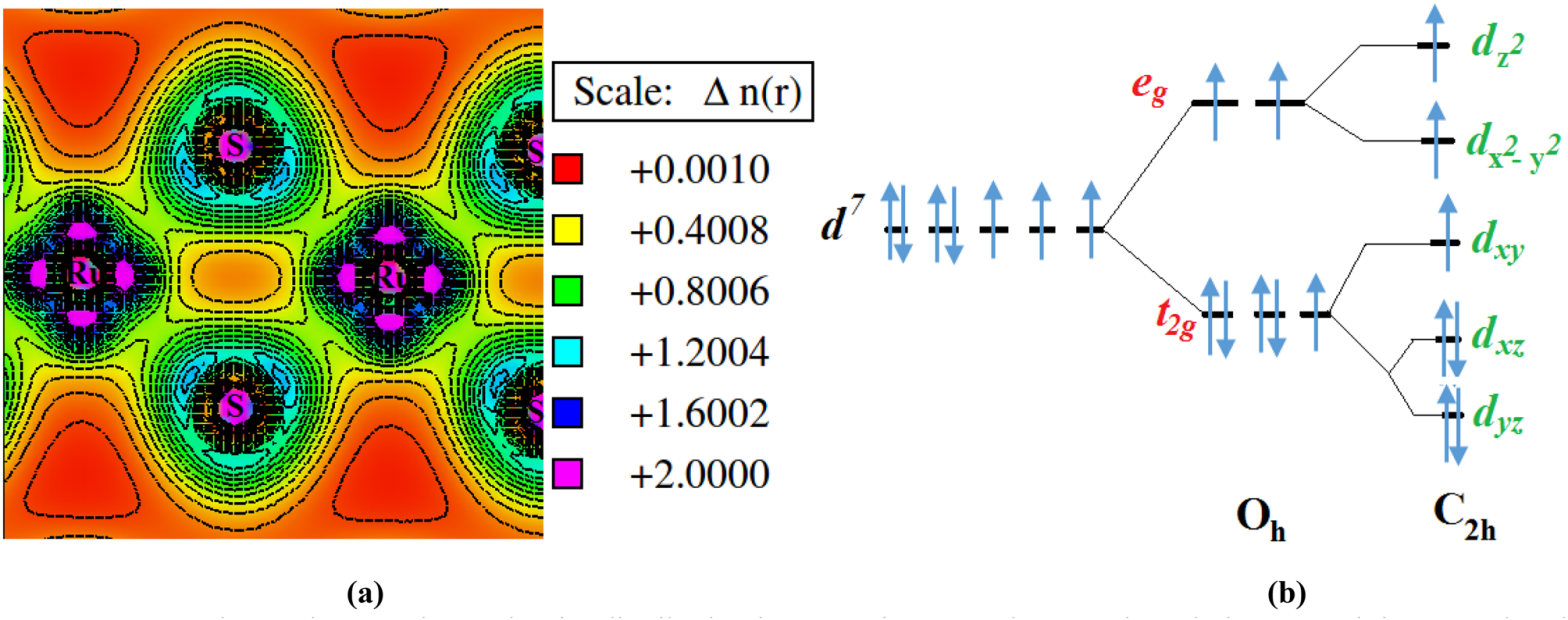
**(a)** Valence electron charge density distribution in marcasite RuS_2_ along a selected plane containing Ru–S bonds ,illustrating high covalent and low ionic bonding. **(b)** Crystal field splitting of Ru-*4d* levels in marcasite RuS_2_ under C_2h_ symmetry.

The nature of the chemical bonding can also be studied from the DOS plots (Fig. 4a) where a strong interacting Ru and S states is behind the origin of both chemical and ionic type of bonding in m-RuS_2_. The strongly hybridized degenerate of Ru-*4d* and S-*3p* states leads to covalent character of this material whereas the low difference in the relative quantity of those states above and below the Fermi energy level (E_F_) induces ionic nature with low degree of ionization. The bonding mechanism can be further understood from the valence electron charge distribution contour plot along a selected plane containing Ru–S atoms (Fig. 5a). The presence of isolines in the charge density contour between Ru and S atoms indicate strong covalent bond character between S–S and Ru–Ru atoms that dominates their ionic nature due to small degree of localization in the charge distribution.

The Hanney-Smyth relation^41^ (Eq. 8) also gives only 11% of ionic nature of bonding between Ru–S and the covalent nature can be expected for the rest.

8 $$ Ionic\,\,character\, = \,0.16\,\left| {\chi_{Ru\,} \, - \,\chi_{S} } \right|\, + \,0.35\,\left| {\chi_{Ru\,} \, - \,\chi_{S} } \right|^{2} $$

where, $\chi_{Ru\,}$ and $\chi_{S\,}$ are the electronegativity of the Ru and S atoms ($\chi_{Ru\,}$ = 2.2 and $\chi_{S}$ = 2.58). The appearance of low ionicity can be explained from the Lӧwdin partial charge analysis^42,43^ based on plane wave pseudo potential method incorporated in Quantum expresso^44^. Lӧwdin charges are correction over Mulliken charges, which suffer from a serious defect of exaggerated charge separation^45^. The results of Lӧwdin charge analysis (Table 8 in SI) are extension to the electron density map and can be used to quantify the charge transfer between Ru and S atoms. The charge transfer from S atom to Ru atom with an electron transfer number of only 0.08 (0.02 × 4)) and thus 89% of bonds are covalent type. This weak ionic character is attributed to the low electronegativity difference between Ru and S atoms. To the best of our knowledge, this type of Lӧwdin charge analysis used to account the bonding and valence electron density distribution of a material is the first ever report and hence no comparison can be made with previous data.

The presence of strong hybridization and the covalent nature of bonding between Ru–S is also responsible for the formation of energy band gap at E_F_ and the projected density of states plot (Fig. 4a) can be utilized to understand the mechanism. The m-RuS_2_ crystal shows octahedral symmetry where the electrostatic octahedral field (*O*_*h*_) causes crystal field splitting of *d*-orbitals into doubly degenerate *e*_g_ and triply degenerate *t*_2g_ states. The RuS_6_ octahedral units in orthorhombic structure share their edges and they are strongly distorted as compared to the pyrite structure, where they share their corners. This leads to formation of chains along orthorhombic *c*-direction causing the Ru-metal complex axial *e*_*g*_-states to experience high repulsion with respect to the S_2_ ligand fields, thereby shifting the *e*_*g*_ states to higher energy levels. The in plane *t*_*2g*_ states experiences a relative low repulsion and are shifted to low energy levels. The trigonal distortion shown by RuS_6_ octahedrons further splits the Ru- *e*_*g*_ and *t*_*2g*_ states, following *C*_*2h*_ symmetry, forming five different *d*_*z2*_*, d*_*x2-y2*_*, d*_*yz*_*, d*_*xy*_ and *d*_*xz*_ states differing in energy (see Fig. 5b). The splitting stimulates S-*3p* states to overlap with the lower edge of the *t*_*2g*_ states and the *C*_*2h*_ symmetry of the transition metals is changed to D_2h_ symmetry, which is a subgroup of octahedral symmetry. Thus, the complex crystal field splitting of the Ru-*d* electrons forms energy levels on overlap of hybrid *d*^*2*^*sp*^*3*^ orbitals of Ru with *sp*^*3*^ hybridized orbital of S. The Fermi level is shifted just above the bonding *t*_*2g*_ states, and the difference in energy level with the *sp*^*3*^ state (which now has higher energy level than *d- t*_*2g*_ state) is observed as band gap. The electrons fully occupy the bonding *t*_*2g*_ state creating a lack of electrons for metallic conduction and hence m-RuS_2_ is a semiconductor.

### Optical absorption

**Figure 6:**
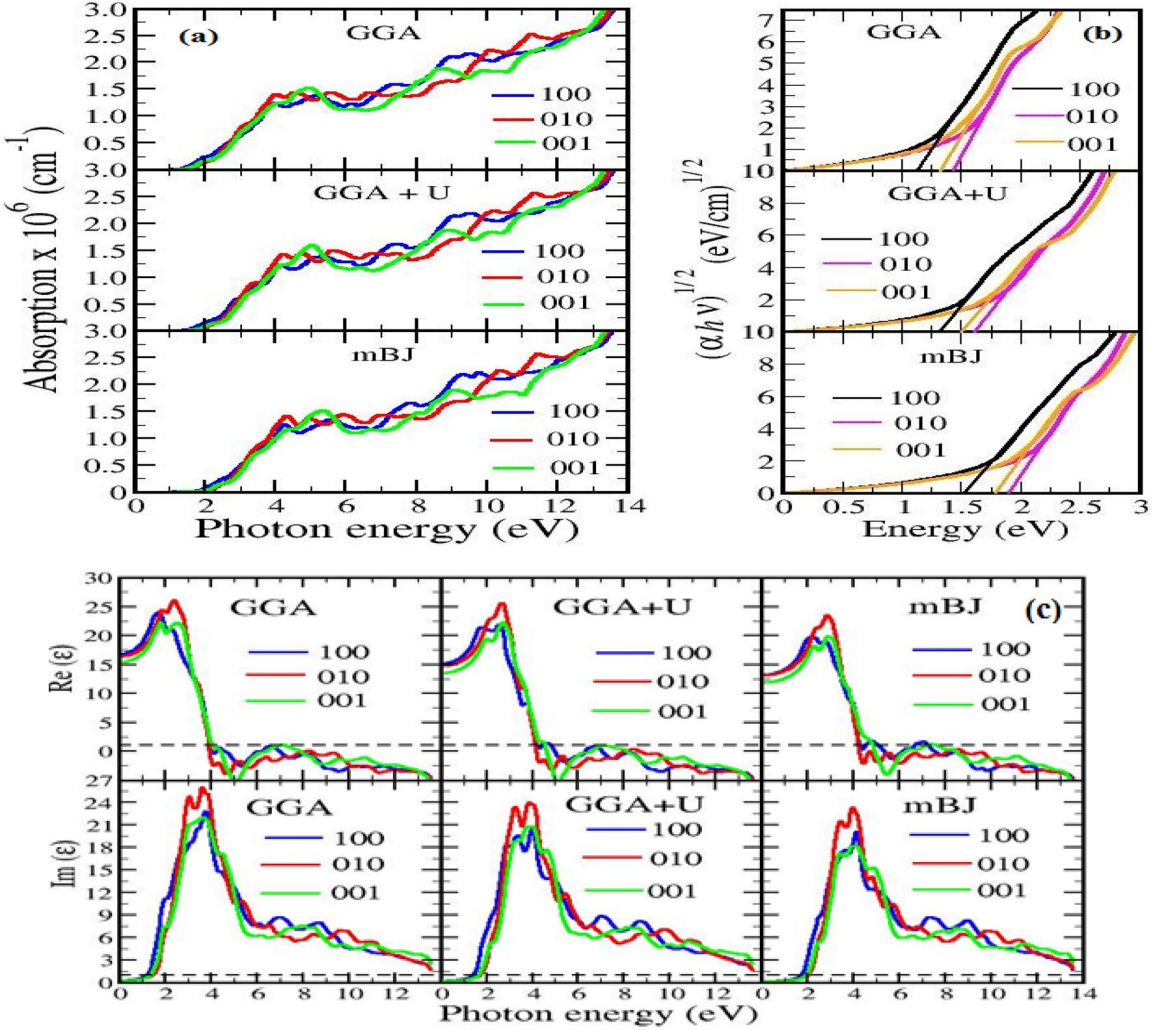
**(a)** Optical absorption coefficient *α* along 100 (blue line), 010 (red line) and 001 (green line) directions plotted under various electron exchange schemes. **(b)** Estimation of optical band gaps from the intercept at (αhѵ)^1/2^ = 0 of (*αhѵ*)^1/2^ vs photon energy (*hѵ*) plot. **(c)** Optical absorption coefficient *α* along 100 (blue line), 010 (green line) and 001 (red line) directions plotted under various electron exchange schemes for Ru doped m-FeS_2_.

For an accurate determination of absorption coefficient, a two particle Green’s function is necessary, as optical excitation accounts for the coupled electron–hole pair created. Thus, to know the uttermost significance of the excitonic effects on optical absorption, a computationally cheap and demanding method based on time dependent DFT serves the purpose. Considering the limitation of this method within the materials of low number of atoms per unit cell, like in m-RuS_2_, the DFT calculated results also acts as a good approximation provided the exitonic effects are also relatively low (which is fulfilled in our case). The optical absorption spectra along the three independent directions 100, 010 and 001 of an anisotropic orthorhombic symmetric unit cell of m-sample obtained from the three different approaches are summarized in Fig. 6. The material shows high absorption coefficient and peaks with varying amplitude with an increasing degree of anisotropy in the photon radiation of ultraviolet radiation. However, an isotropic profile is achieved in the low energy visible region and energy beyond 12 eV. In the higher energy limit of visible region, m-RuS_2_ has favorably enhanced absorption coefficient of 8.5 × 10^5^ cm^−1^ as compared to experimentally determined (~ 2.7 × 10^3^ cm^−1^)^12^ for its pyrite prototype that may potentially bypass its pyrite associate for an efficient photovoltaic production. Also, the order of the absorption coefficient in the visible region is comparable to the FeS_2_ polymorph and outperforms other analog TMDC like FeSe_2_^46^, RuTe_2_, RuSe_2_^47^, MoS_2_, MoSe_2_, WS_2_ and WSe_2_^48^. Meanwhile, the absorption in the ultraviolet region of this orthorhombic crystal dominates the TMDC family and can be used for an excellent protection from ultraviolet radiations.

The effect of intraband transitions is neglected and only the interband transition is considered for the semiconducting material. As identified from the energy band structure, the interband transition between the Ru*4d–*Ru*4d*, Ru*4d*–S*3p*, S *3p*–Ru *4d* and S *3p*–S *3p* dominates the absorption spectra in the visible region. Moreover, the majority of transition between Ru-*4d* rich and S-*3p* rich states and other contributions are due to hybridized *s*, *p* and *d* electrons of Ru and S atoms. In the figure (Fig. 6), the peaks marked as A, A* and B are of particular interest among the several peaks of various amplitudes of optical absorption. The Ru–Ru and the Ru–S transition is significantly responsible for the peak A due to optical anisotropy along 100 and 010 directions. Likewise another peak A* analogous to A is due to optical anisotropy along 001 direction and is slightly shifted towards higher energy region. Similarly, the peak B and other sharp structures beyond B are strongly dominated by 3*p*-S to 4*d*-Ru transition. The optical spectra are also investigated by using different functional predicting band gap values close to pyrite structure, where the peaks A, A* and B are shifted to higher energy region corresponding to enhanced band gap between Ru-*4d* and S-*3p* electronic states. The optical band gap (*E*_*g*_^*opt*^) using Tauc’s relation^49^ (Eq. 21 in supplementary file) is evaluated from the (*αhv*)^1/2^ vs energy plot at (*αhv*)^1/2^ = 0 (Fig. 6b) along the three different crystallographic directions (Table 8 in SI). The size of the gap increases as we move from GGA to GGA + U and mBJ with maxima along 010 directions. Since the optical transition is dominated by transition between Ru*–*Ru, it corresponds to transition in Ru *4d t*_*2g*_ state in the valence band and Ru *4 deg* states in the conduction band, thus giving an optical band gap. On the other hand the electronic band gap is associated with the transition between Ru-*4d t*_*2g*_ to S-*3p* state in the conduction band, which obviously has lower value than Ru *4d–*Ru *4d* transition.

To further investigate the effects of Ru doping on m-FeS_2_, the absorption coefficients for different concentrations of Ru (0.25, 0.5, 0.75) are also studied (Fig. 6c). The absorption coefficients depend on the electronic properties which further rely upon the structure of the system and the configuration of dopant. Therefore, here we have considered all possible positions of doping atom for each concentration and the one with least total energy was chosen for calculation. We found Fe_0.75_Ru_0.25_S_2_ to be the best solar cell material, among all the known TMDC compounds, with absorption of the order of 10^6^ in the visible region. However, considering the structural instability in the material as obtained from phonon dispersion curves, we accredit the best material to Fe_0.5_Ru_0.5_S_2_. The material also shows high absorption of the order of 10^6^ but in far visible region near 3 eV. Among the doped structure, the absorption coefficient varies inversely as Fe_0.75_Ru_0.25_S_2_ > Fe_0.5_Ru_0.5_S_2_ > Fe_0.25_Ru_0.75_S_2_ > RuS_2_. Similar to RuS_2,_ the observed optical band gaps are larger than the electronic gaps this may be due to the optical transition occurs between Ru *4d–*Ru *4d*, whereas, electronic band gap is a result of transition between Ru *4d t*_*2g*_ state to Fe *3d* state in the conduction band. However, due to lack of experimental and theoretical reports on these materials the choice of accurate functional among GGA, GGA + U and mBJ to describe the exact optical properties cannot be concluded. If we limit the shifting of absorption peaks to high energy range, GGA can describe the accurate optical properties here and the absorption coefficient so obtained are best known among the TMDC family for making them highly demanding for commercial photovoltaic applications.

## Conclusion

The new orthorhombic phase of RuS_2_ compound is proposed for efficient photovoltaic production from first principle methods. The feasibility of alloying m-FeS_2_ by Ru to increase the optical absorption is also investigated. The new phase is explored via various stability criterion which shows strong possibility that it is a hidden polymorph of the compound. Phonon dispersion results indicate 25% Ru dopants causes the instability of the corresponding structure while other considered dopant percentage shows no structural instability. The high optical absorption in the marcasite phase is attributed to enhanced optical band gaps investigated from crystal field splitting. The new marcasite phase is investigated for various technological applications and the properties are found to bypass that of pyrite.

## Supplementary Information

Supplementary Information.

## Data Availability

The data for this paper are available from H. J. (himanshuabijoshi09@gmail.com).
