# Author Correction: Modulation of optical absorption in m-Fe_1−x_Ru_x_S_2_ and exploring stability in new m-RuS_2_

**DOI:** 10.1038/s41598-021-95557-8

**Published:** 2021-08-02

**Authors:** H. Joshi, M. Ram, N. Limbu, D. P. Rai, B. Thapa, K. Labar, A. Laref, R. K. Thapa, A. Shankar

**Affiliations:** 1 Condensed Matter Theory Research Lab, Kurseong College, Darjeeling, 734203 India; 2 Department of Physics, St. Josephs College, North Point, Darjeeling, 734103 India; 3 Physical Science Research Centre, Pachhunga University College, Aizawl, Mizoram 796001 India; 4 Physics Department, Faculty of Science, King Saudi University, Riyad, Saudi Arabia; 5 Department of Physics, Mizoram University, Aizawl, 796009 India

Correction to: *Scientific Reports* 10.1038/s41598-021-86181-7, published online 23 March 2021

The original version of this Article contained an error in the spelling of the author A. Laref which was incorrectly given as A. Lareef. The original Article has been corrected.

