# Supplementary material for: Modulation of optical absorption in m-Fe1−xRuxS2 and exploring stability in new m-RuS2
Source: Sci Rep. 2021 Mar 23;11:6601. doi: 10.1038/s41598-021-86181-7 (PMC7987963; doi:10.1038/s41598-021-86181-7)
Supplement: Supplementary file 1 — Supplementary Information. [file 41598_2021_86181_MOESM1_ESM.pdf]

Modulation of Optical Absorption in m-$Fe_{1-x}Ru_xS_2$ and Exploring Stability in New m-RuS_2
(Supplementary Information)

1. Methods

The maximum angular momentum of the atomic orbital basis functions inside MT spheres are expanded upto l_{max}=12. The RMT of different atoms in the unit cell are optimized and the values for FeS_2 are Fe = 2.3 a.u., S = 1.88 a.u. and for RuS_2 are Ru = 2.39 a.u. and S = 1.95 a.u. The energy convergence criterion is achieved with energy cutoff 10^{-5} Ry. Since, compounds with orthorhombic structures have internal coordinates and in our calculations, we have further optimized the structure by minimization of the forces acting on the atoms to account for the internal coordinate parameters u and v at ambient condition. To investigate the mechanical stability of the alloys, elastic stiffness constants are evaluated employing the stress-strain method [1, 2]. The orthorhombic elastic constants are calculated from the second order derivatives of the polynomial fit of energy vs strain at zero strain employing the ortho-elastic package [3]. The thermodynamic parameters are investigated within the framework of quasi-harmonic Debye model implementing the Gibbs2 package [4]. The master input file, used in wien2k code for calculation of electronic structure, acts as the key input to this code.

The lattice constants and band gaps of the $Fe_{1-x}Ru_xS_2$ mixtures were fitted with a quadratic function of the concentration x as

$$P(x)=x\,P(0)+(1-x)\,P(1)-b_p\,x(1-x) \tag{1}$$

where, b_p is the bowing parameter and P the relevant property. If b_p=0, then the alloys obey Vegards rule [5].

To obtain information on the variation of electronic band structure upon doping, the effective mass, m^* was calculated within the framework of parabolic band approximation [6] using the equation

$$m^*=\frac{\hbar^2}{d^2E/dk^2} \tag{2}$$

d^2E/dk^2 was obtained by a second order polynomial fit of the wave vector (k) vs energy (E) as

$$E=ak^2+bk+c \tag{3}$$

The mechanical stability of the alloys were investigated by determining their elastic constants and were obtained in accordance to the Hooke's law, using the relationship between stress and strain as

$$\sigma_i=C_{ij}\,\varepsilon_j \tag{4}$$

For orthorhombic crystals, the bulk and shear modulus can be evaluated in terms of the elastic stiffness coefficient elements C_{ij}'s according to Voigt (V) and Reuss (R) approximation as [7]

$$B_V=\frac{1}{9}(C_{11}+C_{22}+C_{33})+\frac{2}{9}(C_{12}+C_{13}+C_{23}) \tag{5}$$

$$\begin{aligned}B_R=\chi[&C_{11}(C_{22}+C_{33}-2C_{23})+C_{22}(C_{33}-2C_{13})-2C_{33}C_{12}+\\&C_{12}(2C_{23}-C_{12})+C_{13}(2C_{12}-C_{13})+C_{23}(2C_{13}-C_{23})]^{-1}\end{aligned} \tag{6}$$

$$G_V = \frac{1}{15}(C_{11} + C_{22} + C_{33} - C_{12} - C_{13} - C_{23}) + \frac{1}{5}(C_{44} + C_{55} + C_{66}) \tag{7}$$

$$G_R = 15\{4[C_{11}\left(C_{22} + C_{33} + C_{23}\right) + C_{22}(C_{33} + C_{13} + C_{33}C_{12} - C_{12}\left(C_{23} + C_{12}\right) \\ C_{13}(C_{12} + C_{13}) - C_{23}(C_{13} + C_{23})] / \chi + 3(C_{44}^{-1} + C_{55}^{-1} + C_{66}^{-1})\}^{-1} \tag{8}$$

$$\text{where, } \chi = c_{13}\left(c_{12}c_{23} - c_{13}c_{22}\right) + c_{23}\left(c_{12}c_{13} - c_{23}c_{11}\right) + c_{33}\left(c_{11}c_{22} - c_{12}^2\right)$$

The arithmetic mean of the Voigt and Reuss approximation gives the Hill approximation (H), that is

$$B_H = \frac{B_V + B_R}{2} \tag{9}$$

$$G_H = \frac{G_V + G_R}{2} \tag{10}$$

The measure of the stiffness of a solid, related to its elasticity is given by the Young's modulus and Poisson's ratio as

$$Y = \frac{9BG}{(3B + G)} \tag{11}$$

$$\eta = \frac{(3B - 2G)}{2(3B + G)} \tag{12}$$

Replacing G by G_V and G_R in Eqs. (11) and (12), one can calculate the Voigt and the Reuss average of Young's modulus and Poisson's ratio.

To further investigate the stiffness of the alloys, the hardness parameter which indicates the resistance of the physical object against compression was evaluated as

$$HP = \frac{(1 + 2\eta)Y}{6(1 + \eta)} \tag{13}$$

Thermodynamic calculations are performed within the framework of quasi-harmonic Debye model [8]. In the limits of the harmonic approximation, the geometry of the system is independent of temperature and serves as the reason of its incapability to explain thermal expansion. In turn the quasi-harmonic approximation achieves the geometrical dependence on temperature to predict the thermal expansion of the crystal by assuming the dependence of geometry of the crystal to phonon frequencies. The non-equilibrium Gibbs free energy can then be expressed as

$$G(V, P, T) = E(V) + PV + F_{vib}(\Theta_D(V), T) \tag{14}$$

where, E(V) is the energy per unit cell obtained by fitting the equation of state at 0K and volume V, without zero-point vibrational energy, PV represents constant hydrostatic pressure conditions, $\Theta_D(V)$ is the Debye temperature and F_{vib} is the vibrational parameter also known as vibrational free energy within the ambit of empirical Debye

The non-equilibrium Gibbs function is obtained with respect to volume at constant temperature and pressure as-

$$\left[\frac{\partial G^*(V; P, T)}{\partial V}\right]_{P,T} = 0 \tag{15}$$

Using equation (14), the thermal equation of state in terms of the equilibrium volume curve V (T,P) can be obtained.

Thermal properties such as the constant volume heat capacity (C_V), with respect to temperature can now be calculated in relation to equation 14 as,

$$C_v = 3nk_B\left[4D\left(\frac{\Theta_D}{T}\right) - \frac{3\frac{\Theta_D}{T}}{e^{\Theta_D/T}-1}\right] \tag{16}$$

The static energies with respect to volume calculated by means of first principle method are fitted to the third order Murnaghan equation of state [9, 10] as

$$E(V) = E_0 + \frac{9V_0B_0}{16}\left\{\left[(V_0/V)^{2/3}-1\right]^3 B'_0 + \left[(V_0/V)^{2/3}-1\right]^2\left[6-4(V_0/V)^{2/3}\right]\right\} \tag{17}$$

Therefore, the interrelation between pressure (P) and volume (V) can be obtained as

$$P(V) = \frac{3B_0}{2}\left[(V_0/V)^{7/3}-(V_0/V)^{5/3}\right]\left\{1+\frac{3}{4}(B_0^{'}-4)\left[(V_0/V)^{2/3}-1\right]\right\} \tag{18}$$

Hence, we obtain the thermodynamic parameters from quasi-harmonic Debye model at any pressure and temperature using E(V) data at T=0 K and P=0 GPa.

The optical constants are obtained from the frequency dependent dielectric function $\varepsilon(\omega)=\varepsilon_1(\omega)+i\varepsilon_2(\omega)$. The real $\varepsilon_1(\omega)$ and the imaginary $\varepsilon_2(\omega)$ part are related with each other and thus only imaginary component is calculated directly from DFT. $\varepsilon_2(\omega)$ is obtained as the momentum matrices between the occupied and unoccupied electronic eigenstates given by the relation [11]

$$\varepsilon_2(\omega)=\frac{\hbar^2e^2}{\pi m^2\omega^2}\sum_{nn^1}\int_k d^3k\,|\langle\vec{k}\,n|\,\vec{p}\,|\vec{k}\,n^{'}\rangle|^2\left[1-f(\vec{k}\,n)\right]\delta(E_{\vec{k}\,n}-E_{\vec{k}\,n^{'}}-\hbar\omega) \tag{19}$$

where $\vec{p}$ is the momentum operator, $E_{\vec{k}\,n}$ is the eigenvalue of the eigen function $|\vec{k}\,n\rangle$ and $f(\vec{k}\,n)$ is the Fermi distribution function. From Kramers-Kronig relation, the real part of the dielectric function is obtained as

$$\varepsilon_1(\omega)=1+\left(\frac{2}{\pi}\right)\int_0^\infty\frac{\varepsilon_2(\omega^{'})\omega^{'}d\omega^{'}}{\omega^{'2}-\omega^2} \tag{20}$$

All other optical constants follows immediately from the dielectric function and the absorption coefficient is obtained as-

$$\alpha(\omega)=\omega\sqrt{2}\left[\sqrt{\varepsilon_1(\omega)^2+\varepsilon_2(\omega)^2}-\varepsilon_1(\omega)\right]^{1/2} \tag{21}$$

To evaluate the optical band gap (E_g^{opt}) in the compound, we use the Tauc's relation for allowed indirect optical transition using the following equation [12]

$$(\alpha h\nu)^{1/2}=A(h\nu-E_g^{opt}) \tag{22}$$

here, A depends upon transition probability and is a constant. For calculation of absorption coefficient, it is important to use sufficient number of k-points and empty bands. The changes of the optical constants with empty band and k-points number in the matrix element for optical calculation have been tested.

2. Structural parameter bowing and its dependence with pressure

The Bravais representation of the basis atoms with Ru_i (i = 1, 2) and S_j (j = 1,…, 4) in the unit cell are as follows-

$\boldsymbol{B}_{Ru}^1$ = (0, 0, 0) $\boldsymbol{B}_{Ru}^2$ = (1/2, 1/2, 1/2)

$\boldsymbol{B}_S^1$ = (u, v, 0) $\boldsymbol{B}_S^2$ = (-u, -v, 0)

$\boldsymbol{B}_S^3$ = (-1/2-u, v-1/2, 1/2) $\boldsymbol{B}_S^3$ = (1/2+u, 1/2-v, 1/2)

In contrast to the fact that bond strength is inversely proportional to the bond distance, the orthorhombic phase of RuS_2 has weaker bonds in comparison to its pyrite companion and m- FeS_2. As mentioned, due to unavailability of any literature the proposed m-RuS_2 structure is obtained via Ru doping in Fe sites of m-$Fe_{1-x}Ru_xS_2$ in a slowly varying concentration of x (x = 0, 0.25, 0.5, 0.75, 1.0) leading to different structures with lattice constants (Table 1). The deviation of the lattice constants of doped structure from a linear curve has been estimated by appending a bowing parameter (b_p) [13, 14] in the Vegard's rule, which predicts the lattice parameter of a relaxed doped structure by linear interpretation between the parent and final alloys. Ideally, for doped alloy following this rule,$b_p \approx 0$ and in the present case the modified Vegard's rule (equation 1) provides a set of bowing parameters as b_a = -12.8 Å, b_b = 2.08 Å and b_c = -0.086 Å. It can be noted here that the positive and negative deviation of *b*-parameter from zero indicates the downward and upward bowing respectively (Figure 1a). Furthermore, the validity of the parameters so obtained for $Fe_{1-x}Ru_xS_2$ also can be estimated from the dynamical stability in the absence of the available data. Similarly, the *B* and *B*' of m-RuS_2 are further utilized to understand the relative change in the lattice constants with hydrostatic pressure upto 10 GPa (Fig. 1b), where b/b_0 has negligible effect however, a/a_0 and c/c_0 falls sharply with increasing pressure upto 4 GPa, here a_0, b_0, c_0 are lattice constants at zero pressure. Unlike, other orthorhombic alloys, here the curve drops down linearly with constant *b/a* and *c/a* ratios of 1.238 and 0.783, respectively, for further increase in pressure. An unexpectedly slow rise in amplitude of *b* upto 2 GPa and then falls sharply with further increase in pressure also attributes to the downward bowing of the *b*-parameter. The further increase in pressure above 4 GPa, the relative change in lattice parameters *a* and *c* decreases following a linear relation of constant ratio and hence coexist in the plot.

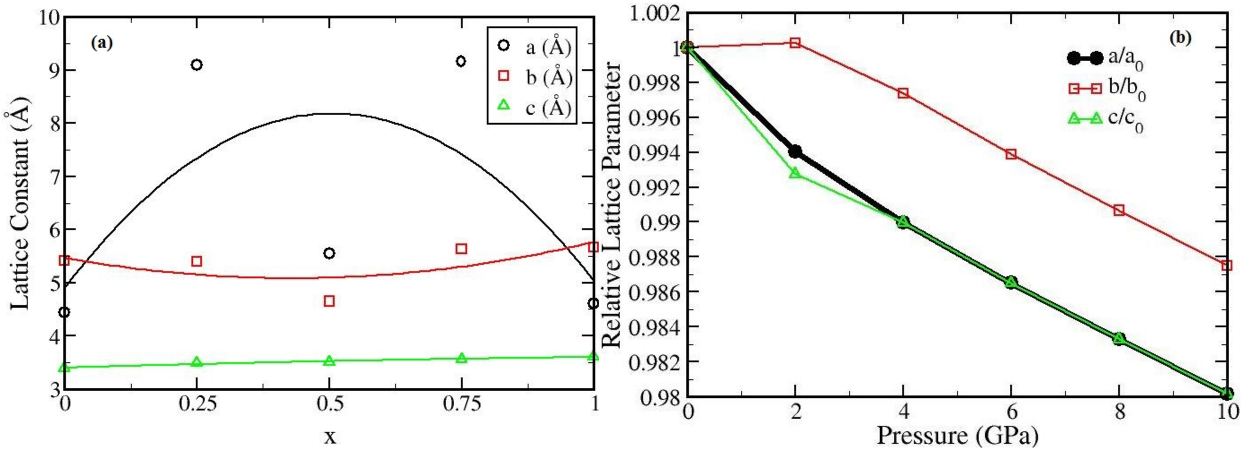

Figure 1: (a) Lattice parameters of Orthorhombic$Fe_{1-x}Ru_xS_2$ plotted as a function of Ru concentration x. The bowing parameters for a, b and c are respectively -12.8 Å, 2.08 Å and -0.086 Å, **(b)** Variation of relative lattice parameters of m-RuS_2 with pressure up to 10 GPa.

Table 1: The lattice constants (*a*, *b* and *c*), internal parameters (*u* and *v*), bulk modulus (*B*), its pressure derivative (*B*′) and bond lengths for *m*-$Fe_{1-x}Ru_xS_2$.

x	***a, b, c* (Å)**	***u, v***	***B* (GPa)**	***B'* (GPa)**	**Fe-Fe/ Ru-Ru/ Ru-Fe (Å)**	**Fe-S (Å)**	**S-S (Å)**

**0**	4.439, 5.415, 3.389<br>4.439, 5.408, 3.388[a]<br>4.436, 5.414, 3.381[b]<br>4.373, 5.381, 3.407[c]<br>4.443, 5.425, 3.387[d]	0.208, 0.375<br>0.206, 0.375[a]<br>0.2,0.378[b]<br>0.203, 0.380[c]	149.6<br>150.1[a]<br>146.5[b]	5.67<br>5.4[a]<br>4.9[b]	3.382 (Fe-Fe)<br>3.38[a]<br>3.36[b]<br>3.386[c]	2.22<br>2.23[a]<br>2.21[b]<br>2.229[c]	2.19<br>2.20[a]<br>2.19[b]<br>2.195[c]
**0.25**	9.095, 5.402, 3.491	0.103, 0.375	132.5	8.4	3.49 (Fe-Fe)<br>3.49 (Ru-Ru)<br>3.94 (Ru-Fe)	2.3	2.31
**0.5**	5.549, 4.651, 3.509	0.205, 0.375	147.7	3.1	3.51 (Fe-Fe)<br>4.02 (Ru-Fe)	2.33	2.36
**0.75**	9.163, 5.632, 3.559	1.03, 0.375	158.6	5.59	3.56 (Fe-Fe)<br>3.56 (Ru-Ru)<br>4.04 (Ru-Fe)	2.31	2.42
**1**	4.607, 5.664, 3.608	0.198, 0.382	198.3	5.3	3.61 (Ru-Ru)	2.37	2.27

[a]Ref 16, [b]Ref. 17, [c]Ref. 15, [d]Ref. 18.

3. Formation Energy

The feasibility of the doping process in m-$Fe_{1-x}Ru_xS_2$ (x=0.25, 0.5, 0.75, 1.0) is verified from their formation energy (E_f) given by the following equation [19]

$$E_f = E_{doped} - E_{pure} + n(E_{Fe} - E_{Ru}) \tag{23}$$

where, E_{doped} is the total energy of the Ru-doped *m*-FeS_2 compound, $E_{pure}$is the total energy of pure *m*-FeS_2, n is the number of Fe atoms replaced by Ru, E_{Fe} & E_{Ru} are the chemical potential of Fe and Ru atoms respectively. Here, while calculating E_f, the chemical potential of Ru (E_{Ru}) is obtained in the bulk structure and meanwhile the chemical potential of Fe (E_{Fe}) depends upon the two extreme material growth condition, such as Fe-rich and S-rich. In Fe-rich growth, E_{Fe}(Fe) is evaluated as the energy per Fe in the bulk Fe system, whereas E_{Fe} (S) is the energy difference between a formula unit of m-FeS_2 and bulk S system in S-rich and out of two conditions, the S-rich growth is much feasible here for the formation of the doped alloy (Table 2).

Table 2: Calculated formation energies ofm-$Fe_{1-x}Ru_xS_2$for different level of Ru concentration under Fe-rich and S-rich conditions.

**x**	**Space Group**	**E_f (eV)**	
		**Fe-rich**	**S-rich**
0.25	P2/m (No. 10)	-0.39	-1.77
0.50	P2/m (No. 10)	-0.78	-3.54
0.75	P2/m (No. 10)	-1.15	-4.86
1.0	Pnnm (No. 58)	-1.30	-7.02

4. Mechanical Stability

The stiffness matrix takes the following form with no relationship with one another-

$$C_{ortho} = \begin{pmatrix} C_{11} & C_{12} & C_{13} & 0 & 0 & 0 \\ 0 & C_{22} & C_{23} & 0 & 0 & 0 \\ 0 & 0 & C_{33} & 0 & 0 & 0 \\ 0 & 0 & 0 & C_{44} & 0 & 0 \\ 0 & 0 & 0 & 0 & C_{55} & 0 \\ 0 & 0 & 0 & 0 & 0 & C_{66} \end{pmatrix} \quad (24)$$

The elastic anisotropy can be understood from the variation of Y, linear compressibility, G and η along the three directions (Figure 2), where the deformation of the spherical surface measures the degree of elastic anisotropy.

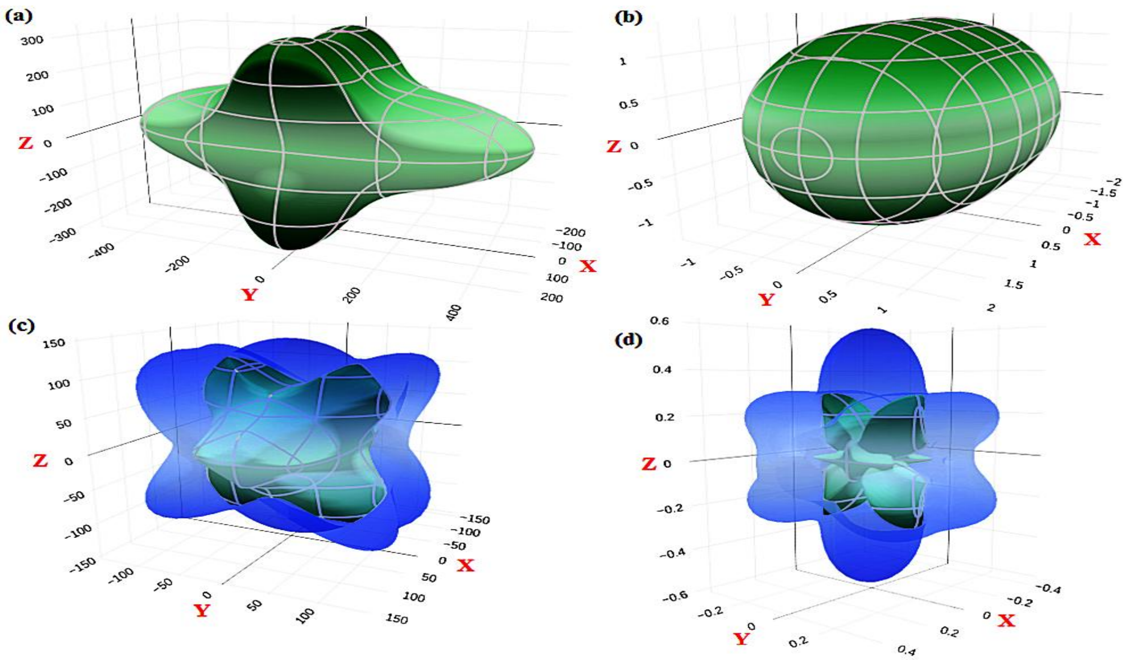

Figure 2: Spatial dependence of **(a)** Young's Modulus, **(b)** linear compressibility, **(c)** shear modulus and **(d)** Poisson's ratio of m-RuS_2.

Table 3: The elastic constants (C_{ij}) in GPa, Bulk moduli (B) in GPa, Shear moduli (G) in GPa, Young's moduli (Y) in GPa, G/B, Poisson ratio (η), Transverse elastic wave velocity (V_t) in m/s, Longitudinal elastic wave velocity (V_l) in m/s, Mean wave velocity (V_m) in m/s, Debye temperature (Θ_D) in K and Hardness parameter (H) in GPa for Ru concentration of 0.25, 0.5, 0.75 and 1.

x	0.25	0.50	0.75	1.0
C_{11}	261.624	373.767	246.918	306.071
C_{22}	311.911	237.013	335.290	507.115
C_{33}	301.939	278.761	321.944	395.033

**C_{44}**	79.675	95.226	63.324	78.046
**C_{55}**	93.888	55.045	111.794	144.993
**C_{66}**	61.802	100.742	100.135	123.811
**C_{12}**	82.195	55.213	35.194	97.192
**C_{13}**	115.352	047.234	144.004	182.095
**C_{23}**	56.708	115.721	60.606	75.591
**B**	153.710	146.730	171.550	201.841
**G**	86.031	89.547	94.318	118.491
**Y**	217.512	223.229	234.339	299.611
**G/B**	0.56	0.61	0.62	0.55
**η**	0.264	0.246	0.242	0.266
**V_t**	4113.86	4138.70	4117.25	4386.96
**V_l**	7266.54	7134.81	7059.77	7750.36
**V_m**	4574.82	4592.84	4566.83	4878.61
**Θ_D**	561.211	553.259	547.564	590.898
HP	13.53	15.16	16.22	18.46

5. Thermodynamic Properties

Constant volume specific heat (C_v) was found to increase linearly with respect to temperatures up to 200 K, almost for all concentrations of Ru. Above this temperature up to 900 K (for x = 0.25) and 1200 K (for x = 0.5, 0.75, 1.0), the linearity decreases with the increase in temperature. C_v then becomes independent of temperature and attains a constant value at all ranges of pressure between 0-20 GPa. At lower temperatures up to 200 K, C_v obeys the Debye's T^3 law, at constant pressure. Also, at higher temperatures C_v obeys the Dulong and Petie's law i.e. it attains a constant value. The temperature variation of the constant volume specific heat shows the validation of two important laws of thermodynamics, thereby, reveling that the alloys remain in solid state for the explored range of temperature and pressure.

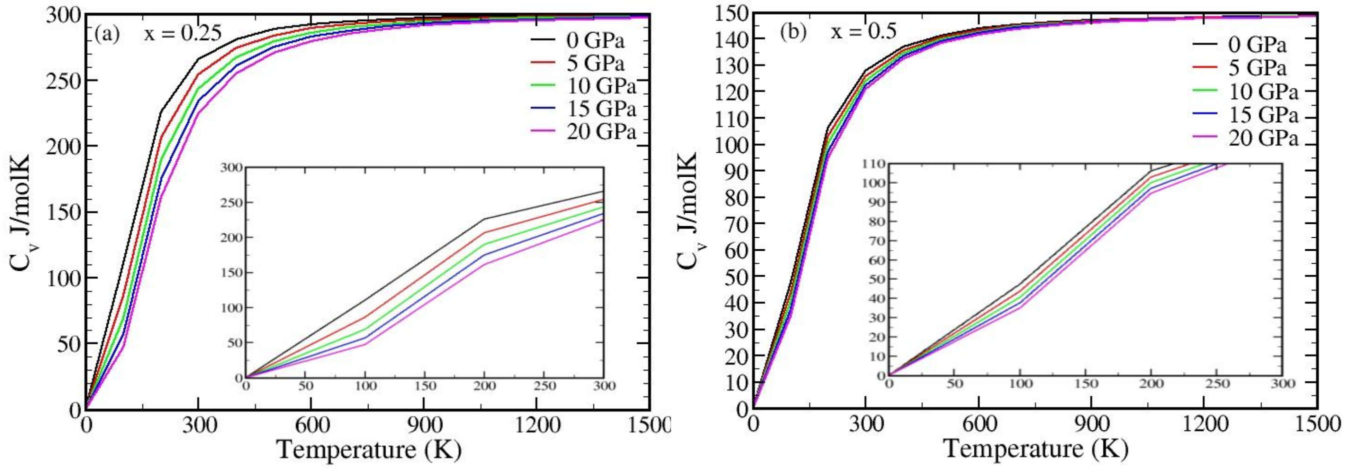

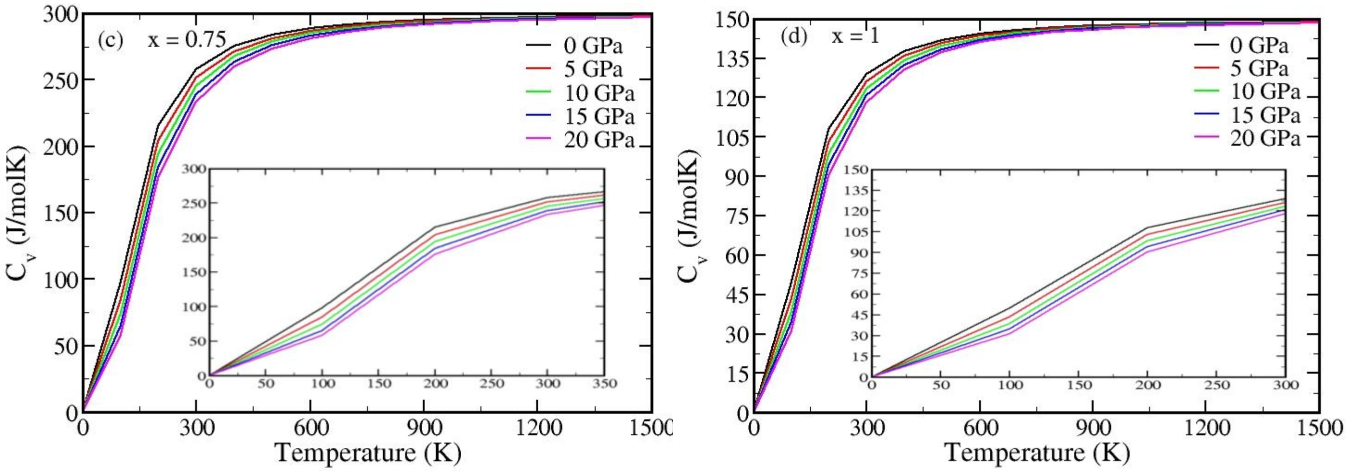

Figure 3: Constant volume specific heat capacity as a function of temperature at a pressure range of 0 to 20 GPa for Ru concentration of **(a)** x = 0.25 **(b)** x = 0.5 **(c)** x = 0.75 and **(d)** x = 1. The insert shows the range of low temperatures for which the materials obey Debye's T^3 law.

6. Dynamical Stability

The irreducible representation of optical vibrations in an orthorhombic symmetry at Γ-point is expressed as-

$$\Gamma = 2A_g + 2B_{1g} + B_{2g} + B_{3g} + 2A_u + B_{1u} + 3B_{2u} + 3B_{3u} \quad (25)$$

In m-RuS_2, six atoms per unit cell corresponds to 18 phonon modes of which six R-active and seven IR- active optical phonon modes, three are acoustic modes, whereas A_u mode is inactive. Note that the A's and the B's are singly degenerate modes and due to inversion symmetry of RuS_2 crystal, the R and IR modes are mutually exclusive modes. The inactively considered A_u mode in fact gives symmetric nature of optical branches in a careful analysis, that points towards the hyper-Raman activity and in addition to this B_{3u}, B_{2u} *and* B_{1u} also show hyper-Raman activity. The characteristic activities of different symmetries have been listed in Table 4 and the zone centered frequencies in Table 5. The R-activity modes arise entirely from the atom at *4g* Wyckoff position of the unit cell, i.e., S atom, whereas the IR and hyper-Raman active modes are due to Ru and S atoms together. The optical and acoustic modes of vibration are overlapping to each other and hence no phonon band gap is opened up. A similar feature is also visible in phonon density of states where the optical phonons are consist of longitudinal (LO) and transverse optical (TO) modes with wave vectors parallel and perpendicular to atomic displacement, respectively. Here, almost all the observed optical vibrations are out-of- phase with neighboring lattice atoms and therefore the strong and weak coupling between them gives an interesting feature of separate high and low frequency optical branches. The high frequency optical modes correspond to phonon-photon coupling and the low frequency modes to reststrahlen band. This band is located in an interval between TO - LO phonon frequencies also known as reststrahlen frequency (52.9 cm^{-1}), where the real part of the dielectric constant is negative and the reflectivity of the material approaches to unity. At a frequency just above the first LO frequency (ω_L), when a screening parameter $\varepsilon(\infty) > 1$ is introduced to represent absorption at high frequencies the reflectance corresponds to a sharp minimum. Then, two limiting parameters of reststrahlen band can be related by Lyddane-Sachs-Teller relation [20] as $(\omega_L^2 / \omega_T^2) = \varepsilon_0 / \varepsilon(\infty)$,which gives the ratio of static and high frequency dielectric constants. The marcasite phase, highly polar natured with larger reststrahlen band, is also a potential candidate for optoelectronic devices over pyrite phase. The $\omega_{T,}$ parameter is also a measure of hardness index and correlates to the melting temperature (T_m). The parameter is obtained by considering a number of melting temperature data [21] of various compounds and applying the best single power fit method of Ribbibg et al. [22], which gives the relation Tm=10400 $\{\lambda T(\mu m)\}^{-0.52}$ and the estimated values are summarized in Table 6.

Table 4: Activity of different mode symmetries in orthorhombic RuS_2. The numerical value indicate the number of modes undergoing the activity.

	A_g	A_u	B_{1g}	B_{1u}	B_{2g}	B_{2u}	B_{3g}	B_{3u}
**Infrared**	x	x	x	2	x	4	x	4
**Raman**	2	x	2	x	1	x	1	x
**Hyper-Raman**	x	2	x	2	x	4	x	4

Table 5: The optical mode phonon frequencies $(cm)^{-1}$ at q=0 in m and p-RuS_2.

	**m-phase**					**p-phase**	
**Mode**	**symmetry**	**activity**	**frequency**	**Mode**	**symmetry**	**activity**	**frequency**
4	A_u	In.*	173.5	4	A_u	In.	192.5
5	B_{3u}	IR$	194.3	5-7	T_u	IR	207.2
6	B_{2u}	IR	226.4	8-9	E_u	In.	212.8
7	B_{3u}	IR	294.8	10-12	T_u	IR	241.7
8	B_{2u}	IR	328.2	13-15	T_u	IR	319.8
9	A_g	R&	347.8	16	A_u	In.	338.3
10	B_{3u}	IR	355.9	17-19	T_g	R	352.8
11	B_{2u}	IR	372.1	20	A_g	R	359.2
12	B_{2g}	R	377.2	21-23	T_u	IR	364.2
13	A_u	In.	379.8	24-25	E_g	R	370.1
14	B_{3g}	R	387.1	26-27	E_u	In.	374.2
13	B_{1u}	IR	407.8	28-30	T_g	R	387.8
16	A_g	R	414.0	31-33	T_u	IR	401.1
17	B_{1g}	R	423.2	34-36	T_g	R	432.9
18	B_{1g}	R	481.9				

*Inactive, $ infrared active, & Raman active

Table 6: Calculated reststrahlen band, LST relationships, melting temperature and ZPE of orthorhombic and pyrite RuS_2

	**Reststrahlen band (cm^{-1})**	**Reststrahlen wavelength (μm)**	$\varepsilon_0/\varepsilon_\infty$	T_m **(K)**	**ZPE**
**Pyrite**	20.3	492.6	1.222	1333.3	0.160
**Orthorhombic**	52.9	189.03	1.702	1263.2	0.124

7. Electronic Structure

The effect of choice of different exchange correlation potential is distinct in the energy band gaps (E_g) (Table 7). In the DOS plot (Figure 4), the valence region can be divided into two main segments, viz. the deep region between -5.0 to -2.5 eV, which mainly originates from S –*3s* states and the shallow region ranging from -2.4 to 0 eV, dominated by transition metal-*d* state. The transition metal *d*-states is also extended in the conduction region above Fermi energy level (E_F) leaving a sharp peak DOS. The empty states between the valence and the conduction domain correspond to semiconducting nature with gap formation as listed in Table 7. The implementation of advanced functional does not alter the contributions of different electronic states besides the shifting of the valence and conduction band edges and blunt amplitude of the DOS peaks for all concentrations of Ru. The equivalent energy band structures along the high symmetric directions of the first Briollouin zone (Figure 6) where the flat bands along the high symmetrical *Z-T* direction corresponds to sharp peaks in the shallow valence region. Similarly, the prominent flat band along *Y-Γ* direction in the low conduction region of $Fe_{0.75}Ru_{0.25}S_2$ arises due to sharp DOS characteristic observed after the energy gap which is pushed towards the *Γ-X* direction in $Fe_{0.5}Ru_{0.5}S_2$. The broad DOS arising after the gap in case of x=0.75 and 1.0 makes this feature absent in their band structure. The valence and the conduction regions do not overlap thus creating a gap in the allowed energy levels observed as band gap. The DOS figure also shows the contribution of different atoms to the total DOS and is thus clear that the maximum of the valence band and the lowest of the conduction bands both results from the unfilled *d*-states of the transition atom (except for x=1). The observed DOS characteristics are closely relatable to those of $FeSe_2$ but differs from $FeTe_2$ where the valance bands are reported to cross the Fermi region showing semi-metallic nature under GGA scheme [23]. The energy band gaps varies as E_g (x=0.25) >E_g (x=1.0) >E_g (x=0.5) >E_g (x=0.75) and the inclusion of mBJ widens the band gaps almost double whereas energy gap bowing parameter is lowest under GGA+U scheme (Figure 5). Here, the inverse relation of E*g* with Ru concentration (except for x=1.0) is within the conventional knowledge of inversely dependent of band gap of a semiconductor with heavier dopant atom. The bowing parameter obtained using Vegard's law of 1.0 eV is considerable and one can ascertain Ru as an efficient dopant to modulate the electronic structure of m-FeS_2. The energy band structure of x=0.25 is distinguished by presence of an indirect band gap along *T-Γ* direction. The effective mass in the neighborhood of valence band maximum (VBM) and conduction band minimum (CBM) are also calculated (as shown in Table 7) to explore further the difference of electronic structures for different concentrations of Ru. One can note from Table 7 that the RuS_2 bands are highly dispersed with low effective mass as compared to other sample alloys that indicates the presence of strong orbital interaction. Likewise, the absence of flat bands in the neighborhood of band edges also justifies the low effective mass of this material. Such materials are of new trends in thermoelectric devices leading to high conversion efficiency [24].

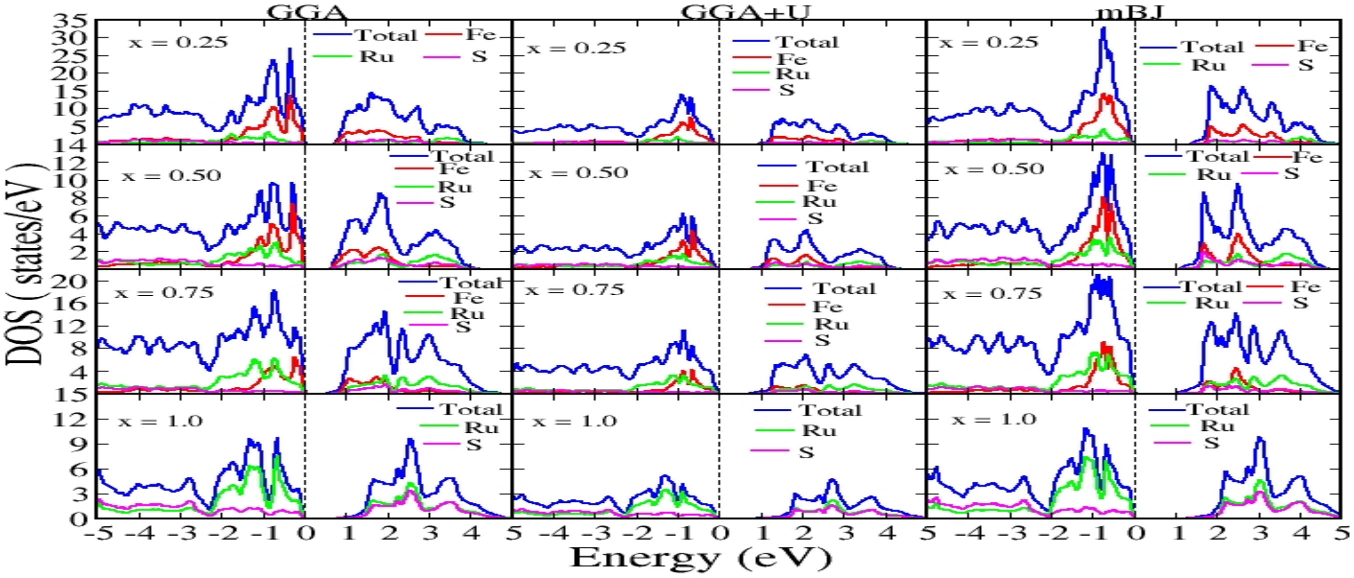

Figure 4: Total density of states along with different atomic contribution for different concentration of Ru. The calculated DOS with GGA, mBJ and GGA+U are shown alongside.

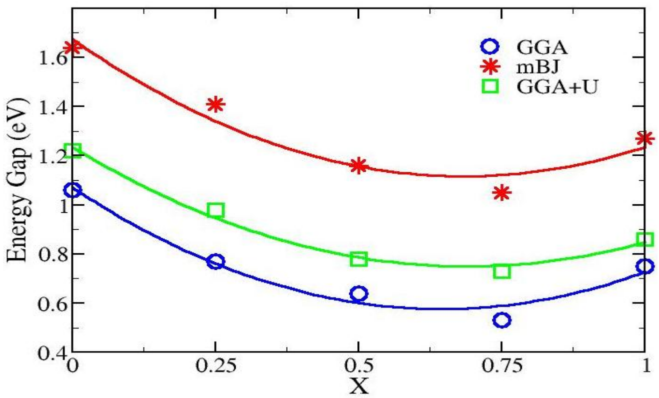

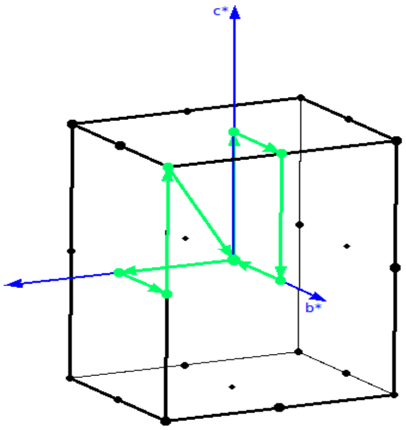

(a) (b)

Figure 5: **(a)** Band gap of $Fe_{1-x}Ru_xS_2$ as a function of concentration x. The bowing parameters under GGA and mBJ schemes are each 1.18 eV and under GGA+U scheme is 1.0 eV. **(b)** The first Brillouin zone selected for band structure calculations in the marcasite alloys.

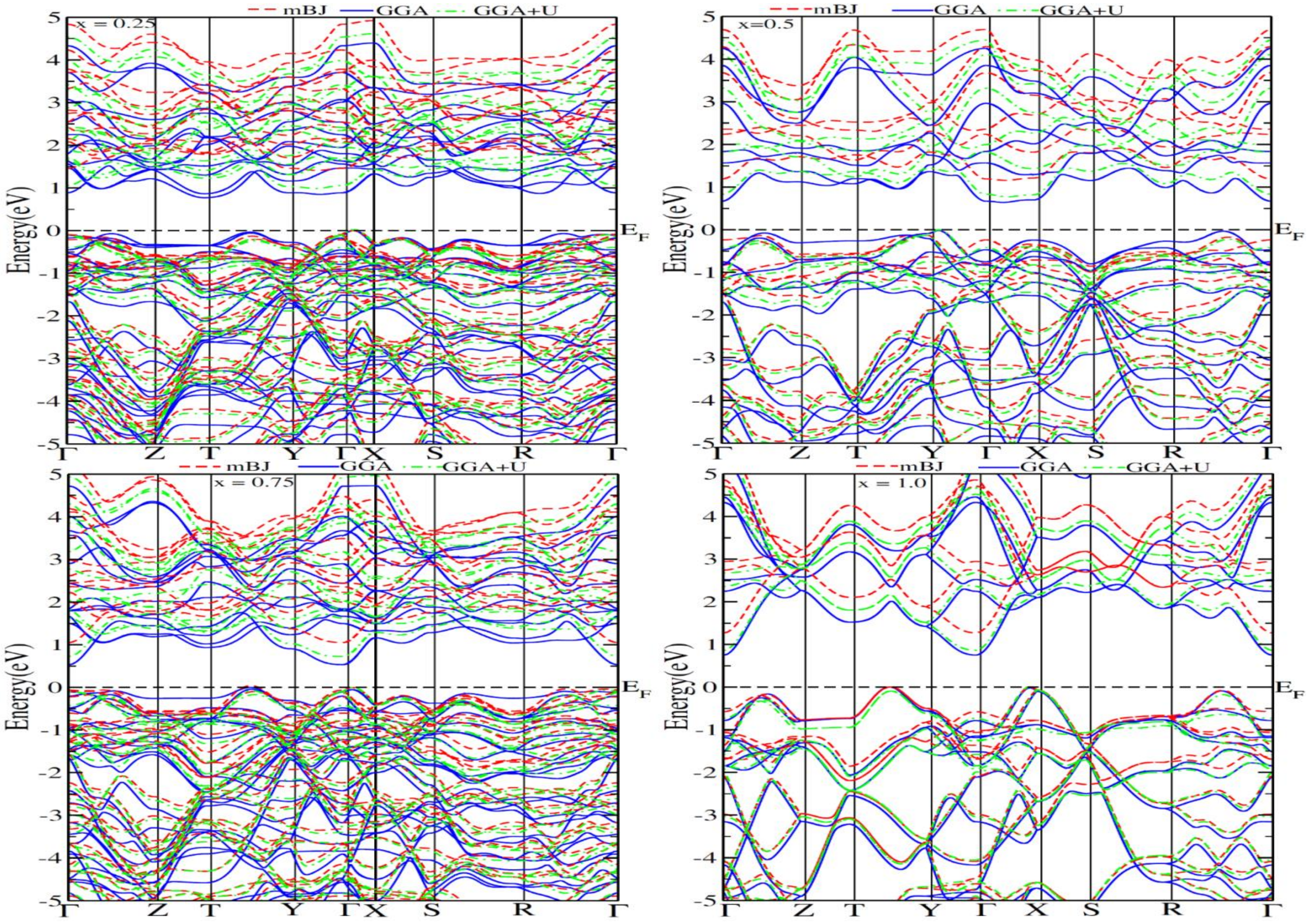

Figure 6: Band structure plotted along high symmetry directions for different concentration of Ru dopants. The red, blue and green lines respectively denotes band structure under mBJ, GGA and GGA+U functionals.

Table 7: Energy band gap (E_g) values in eV and effective mass of the band edges in units of electron rest mass.

x	**scheme**	$\mathbf{E_g}$	**effective mass**	
			**conduction band**	**valence band**
0.25	GGA	0.77	1.299	0.387
	mBJ	1.41	0.939	0.559
	GGA+U	0.98	0.709	0.434
0.50	GGA	0.64	2.447	0.982
	mBJ	1.16	2.917	0.472
	GGA+U	0.78	1.972	0.411
0.75	GGA	0.53	0.405	0.479
	mBJ	1.05	0.479	0.492
	GGA+U	0.73	0.445	0.408
1.0	GGA	0.75	0.382	0.272
	mBJ	1.3	0.426	0.326
	GGA+U	0.86	0.386	0.289

Table 8: Löwdin charge analysis of marcasite RuS_2.

**Species**	***s***	***p***	***d***	**total**	**Charge (e)**
Ru	2.52	5.99	7.22	15.74	-1.74
S	1.74	4.24	0.00	05.98	0.02

8. Optical Absorption

The optical absorption is also closely related to the complex dielectric function *ε(ω)* and their estimated values are presented in Figure 7, where the real part $\varepsilon_1(\omega)$ is obtained from its imaginary part $\varepsilon_2(\omega)$ using the Kramers-Kronig relation (equations 18 and 19). The static dielectric function $\varepsilon_1(0)$ at zero frequency limit (Table 9) is independent of lattice vibration and the high values also denote the advantage for photovoltaic application with strong energy storage capacity. The $\varepsilon_1(0)$value so obtained is in close agreement with experimental optical spectra of p-RuS_2 along 100 direction [12]. However, like other analogous materials [25-27], the $\varepsilon_1(0)$ decreases when GGA+U is considered for calculation and further reduces with choice of mBJ. The imaginary part of dielectric function $\varepsilon_2(\omega)$ is also related to energy dissipation via absorption and the two major peaks one at the far visible region at energy about 3.0 eV and another at the near ultraviolet region at energy 3.8 eV is identical to optical absorption. The transition and the orbital contribution to the peaks are similar to the absorption and the peaks are also shifted to higher energy range with choice of exchange correlation functional.

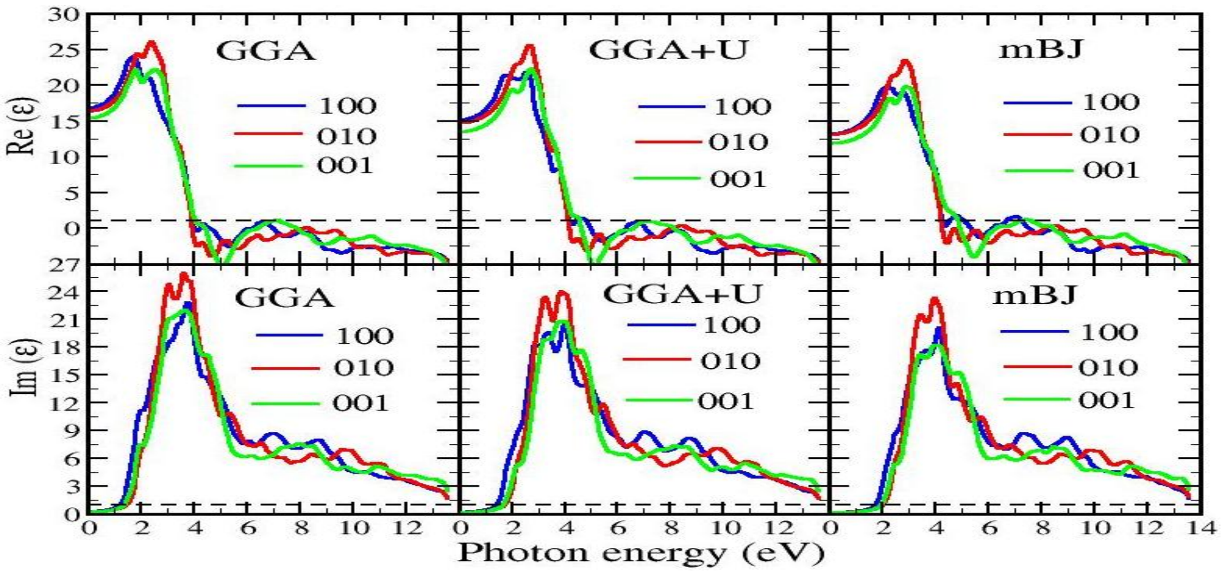

Figure 7: Dielectric function *ε(ω)* of RuS_2 marcasite calculated with GGA, GGA+U and mBJ. The dashed black line indicates the vacuum dielectric constant of 1.

Table 9: Optical band gap E_g^{opt} (eV) and static dielectric function $\varepsilon_1(0)$ of pure and doped structures.

x	scheme		E_g^{opt}			$\varepsilon_1(0)$	
		**100**	**010**	**001**	**100**	**010**	**001**
0.25	GGA	0.91	0.95	1.06	22.4	21.7	18.7
	GGA+U	1.30	1.43	1.43	18.6	16.3	14.1
	mBJ	1.73	1.81	1.81	14.6	13.8	11.4
0.50	GGA	1.03	0.86	0.87	21.0	23.5	18.6
	GGA+U	1.30	1.15	1.30	16.7	18.9	14.4
	mBJ	1.71	1.80	1.80	13.8	15.2	11.6

0.75	GGA	0.90	1.02	1.10	20.4	19.7	17.4
	GGA+U	1.18	1.42	1.42	17.7	16.8	14.4
	mBJ	1.74	1.84	1.84	14.9	14.2	12.3
1.0	GGA	1.15	1.45	1.35	17.1	16.6	15.4
	GGA+U	1.33	1.62	1.50	15.8	14.8	13.5
	mBJ	1.54	1.91	1.80	13.2	13.1	11.9

Reference

[1] Dzade, N. Y. & de Leeuw, N. H. Periodic DFT+U investigation of the bulk and surface properties of marcasite (FeS2). *Phys. Chem. Chem. Phys*., **19**, 27478 (2017).

[2] Page, Y. L. & Saxe, P. Symmetry-general least-squares extraction of elastic data for strained materials from ab initio calculations of stress. *Phys. Rev. B*, **65**, 104104 (2002).

[3] Reshak, A. H. & Jamal, M. DFT calculation for elastic constants of orthorhombic structure within WIEN2Kcode: A new package (ortho-elastic). *Journal of Alloys and Compounds*, **543**, 147–151 (2012).

[4] Otero-de-la-Roza, A., Abbasi-Pe´rez, D. & Luana, V. Vibrational and Thermal Properties of Oxyanionic Crystals. *Comput. Phys. Comm.*, **182**, 2232 (2011).

[5] Vegard, L. Die Konstitution der Mischkristalle und die Raumfüllung der Atome. *Z. Cryst.*, **67**, 239 (1928).

[6] Wang, L. L. *et al.* Thermoelectric Performance of Half-Heusler Compounds TiNiSn and TiCoSb. *Journal of Applied Physics*, **105**, 013709 (2009).

[7] Ravindran, P., Fast, L., Korzhavyi, P. A., & Johansson, B. Density functional theory for calculation of elastic properties of orthorhombic crystals: Application to $TiSi_2$. *J. Appl. Phys.*, **84**, 4891 (1998).

[8] Blanco, M. A., Francisco, E. & Luana, V. GIBBS: isothermal-isobaric thermodynamics of solids from energy curves using a quasi-harmonic Debye model. *Comput. Phys. Comm.*, **158**, 57 (2004).

[9] Murnaghan, F. D. The Compressibility of Media under Extreme Pressures. *Proc. Natl. Acad. Sci.*, USA, **30**, 5390 (1994).

[10] Qiang, L., Duo-Hui, H., Qi-Long, C. & Fan-Hou, W. Phase transition and thermodynamic properties of $BiFeO_3$ from first-principles calculations. *Chin. Phys. B*, **22** (3), 037101 (2013).

[11] Saha, S., Sinha, T. P. & Mookerjee, A. Electronic structure, chemical bonding, and optical properties of paraelectric $BaTiO_3$. *Phys. Rev. B*, **62**, 8828–8834 (2000).

[12] Tauc, J. Optical properties and electronic structure of amorphous Ge and Si. *Materials Research Bulletin*, **3**, 37–46 (1968).

[13] Liou, B. T., Yen, S. H. & Kuo, Y. K. Vegard's law deviation in band gap and bowing parameter of $Al_xIn_{1-x}N$. *Appl. Phys. A*, **81**, 651-655 (2005).

[14] Darakchieva, V. *et al.* Lattice parameters, deviations from Vegard's rule, and E2 phonons in InAlN. *Appl. Phys. Lett.*, **93**, 261908 (2008).

[15] Sithole, H. M., Nguyen-Manh, D., Pettifor, D. G. & Ngoepe, P. E. Internal Relaxation, Band Gaps and Elastic Constant Calculations of FeS_2. *Mol. Simul.*, **22**, 31−37 (1999).

[16] Gudelli, V. K., Kanchana, V., Appalakondaiah, S., Vaitheeswaran, G. & Valsakumar, M. C. Phase Stability and Thermoelectric Properties of the Mineral FeS_2: An Ab Initio Study. *J. Mater. Chem. C*, **117**, 21120-21131 (2013).

[17] Buerger, M. J. Zeitschrift Fuer Kristallographe, Kristallgeometre, Kristalliphysik, 97A, 504 (Pearson handbook of crystallographic data.) (Kristallchemie 1937).

[18] Sun, R., Chan, M. K. Y. & Ceder, G. First-principles electronic structure and relative stability of pyrite and marcasite: Implications for photovoltaic performance. *Phys. Rev. B*, **83**, 235311 (2011).

[19] Zeng, Z. H., Vallejo, F. C., Mogensen, M. B. & Rossmeisi, J. Generalized trends in the formation energies of perovskiteoxides. *Phys. Chem. Chem. Phys*., **15**, 7526-7533 (2013).

[20] Lyddane, R. H., Sachs, R. G. & Teller, E. On the Polar Vibrations of Alkali Halides. *Phys. Rev.*, **59**, 673 (1941).

[21] Dobrowolski, J. Handbook of Optics (ed. W. Driscoll and S. Vaughan), Chap. 8, Section 99 (McGraw-Hill, 1995).

[22] Ribbibg, C. G. & Wakelgard, E. Reststrahlen bands as property indicators for materials in dielectric coatings. *Thin Solid Films*, **206**, 312-317 (1991).

[23] Schena, T. First-Principles Study on Pyrites and Marcasites for Photovoltaic Application. *Energy & Environment*, **254** (2015).

[24] Pei, Y., LaLonde, A. D., Wanga, H. and Snyder, G. J. Low effective mass leading to high thermoelectric performance. *Energy Environ. Sci.*, **5**, 7963-7969 (2012).

[25] Ganga, B. G., Ganeshraj, C., Krishna A. G. & Santhosh, P. N. Electronic and optical properties of $FeSe_2$ polymorphs: solar cell absorber. Preprint at https://arxiv.org/abs/1303.1381 (2013).

[26] Yang, T. R., Huang, Y. S., Chyan, Y. K. & Chang, J. D. Proceedings of the 21st International Conference on Low Temperature Physics, Prague, August 8-14, (1996).

[27] Tang, W., Rassay, S. S., & Ravindra, N. M. Electronic & Optical properties of Transition-Metal Dichalcogenides. *Madridge J Nanotechnol Nanosci.*, **2**(1), 58-64 (2017).
